# Genome interpretation using in silico predictors of variant impact

**DOI:** 10.1007/s00439-022-02457-6

**Published:** 2022-04-30

**Authors:** Panagiotis Katsonis, Kevin Wilhelm, Amanda Williams, Olivier Lichtarge

**Affiliations:** 1 Department of Molecular and Human Genetics, Baylor College of Medicine, One Baylor Plaza, Houston, TX 77030 USA; 2 Graduate School of Biomedical Sciences, Baylor College of Medicine, One Baylor Plaza, Houston, TX 77030 USA; 3 Department of Biochemistry, Human Genetics and Molecular Biology, Baylor College of Medicine, One Baylor Plaza, Houston, TX 77030 USA; 4 Department of Pharmacology, Baylor College of Medicine, One Baylor Plaza, Houston, TX 77030 USA; 5 Computational and Integrative Biomedical Research Center, Baylor College of Medicine, One Baylor Plaza, Houston, TX 77030 USA

## Abstract

Estimating the effects of variants found in disease driver genes opens the door to personalized therapeutic opportunities. Clinical associations and laboratory experiments can only characterize a tiny fraction of all the available variants, leaving the majority as variants of unknown significance (VUS). In silico methods bridge this gap by providing instant estimates on a large scale, most often based on the numerous genetic differences between species. Despite concerns that these methods may lack reliability in individual subjects, their numerous practical applications over cohorts suggest they are already helpful and have a role to play in genome interpretation when used at the proper scale and context. In this review, we aim to gain insights into the training and validation of these variant effect predicting methods and illustrate representative types of experimental and clinical applications. Objective performance assessments using various datasets that are not yet published indicate the strengths and limitations of each method. These show that cautious use of in silico variant impact predictors is essential for addressing genome interpretation challenges.

## Introduction

### The need of estimating variant impact

The drastic reduction in the cost of genome sequencing over the last decade (DNA Sequencing Costs: Data 2020) led to a proliferation of large-scale next-generation sequencing (NGS) datasets (Pereira et al. 2020). The NCBI dbGaP database (Mailman et al. 2007) hosts a vast collection of genotype–phenotype data in a common repository, but many other data sets of broad interest reside elsewhere and not all have simple access (Gutierrez-Sacristan et al. 2021). Currently, dbGaP contains more than 500 NGS case–control studies, including studies of such diseases as Alzheimer's (Beecham et al. 2017), Parkinson's (Rosenthal et al. 2016), autism spectrum disorder (ASD) (Fischbach and Lord 2010), and others (Taliun et al. 2021). Additional case–control studies on the same or similar traits are available through other independent initiatives (Marek et al. 2018; Petersen et al. 2010). Other sources, called biobanks, contain sequencing data and clinical diagnostics from individuals representing a population, without a focus on any particular disease. Examples include the UK Biobank, which currently holds genetic data from about 500,000 individuals (Backman et al. 2021; Bycroft et al. 2018), as well as the All of Us Research Program (All of Us Research Program et al. 2019) among others (Wichmann et al. 2011). Some datasets focus on ethnic differences, such as The 1000 Genomes Project (The 1000 Genomes Project Consortium 2015) and various population-specific cohorts (Genome of the Netherlands Consortium 2014; Jeon et al. 2020; Kim et al. 2018; Nagasaki et al. 2015). Each of these sources contains rich data on human genetic variations over which we can address questions of disease etiology for mechanistic understanding, risk for prevention and early screening, personalized therapy for precision medicine, epistatic effects for complex epidemiology, pharmacogenomics for patient stratification, and ethnic diversity for health equity. Accurate metrics for the impact of individual variants is critical to guide answers to these questions.

There are many types of genetic variants, broadly grouped by the region in which the variant occurs and the number of nucleotides affected. Variants can occur in protein coding regions or non-coding regions of the genome. Although the protein coding region represents only 1.2% of the human genome (Encode Project Consortium 2012), past variant interpretation efforts focused on these variants due to their effects in protein synthesis. However, a large proportion of the non-coding genome is functional and harbors variants that drive diseases by influencing regulatory regions controlling gene expression and untranslated regions affecting mRNA translation (French and Edwards 2020). Variants can also encompass single or multiple nucleotides. Substitutions that add or remove nucleotides are called insertions or deletions, respectively, and they are rare compared to single nucleotide variants (SNVs) that account for about 90% of the variants (1000 Genomes Project Consortium et al. 2010). Protein coding insertion and deletion (indel) variants may be pathogenic when they shift the reading frame of mRNA or map to functionally important sites (Lin et al. 2017; Mullaney et al. 2010). Protein coding SNVs may truncate the protein (stop gain and start loss), cause no change to the protein sequence (synonymous), or alter one amino acid (non-synonymous/missense). Stop gain and start loss variants exert profound effects on protein function, resulting in strong selection against them (Bartha et al. 2015). Synonymous variants are assumed to be benign, although they can cause various pre-translational changes (Zeng and Bromberg 2019) and affect codon usage bias (Plotkin and Kudla 2011). The impact of non-synonymous variants is challenging to predict, since they may affect a number of protein characteristics, such as folding (Wang and Moult 2001), protein interactions (Teng et al. 2009), dynamics (Uversky et al. 2008), post‐translational modifications (Yang et al. 2019), solubility (Monplaisir et al. 1986), and others (Stefl et al. 2013), with approximately 30% of variants having a strong impact (Chasman and Adams 2001). Purifying selection accounts for all the above effects and reduces diversity within species (Cvijovic et al. 2018), allowing only beneficial and nearly neutral variants to spread and become fixed (Fu and Akey 2013; Patwa and Wahl 2008). Assuming the variant effects differ little between homologous proteins, the genetic differences between the species offer valuable information for estimating the overall effect of a variant (Ng and Henikoff 2001). The 3D structures of proteins can also provide additional complementary insights (Orengo et al. 1999; Ramensky et al. 2002). Consequently, homology and structure information have been the two main types of input for estimating the effects of coding variants on protein function.

### Available methods for predicting variant effects

Many computational methods estimate variant effects (Hu et al. 2019), but their aims may differ. Some methods focus on specific aspects of protein function, such as folding stability of the mutated protein (Capriotti et al. 2005, 2008; Cheng et al. 2006; Dehouck et al. 2011; Fariselli et al. 2015; Guerois et al. 2002; Parthiban et al. 2006; Pires et al. 2014; Quan et al. 2016; Worth et al. 2011; Zhou and Zhou 2002) or a combination of folding stability and binding affinity (Berliner et al. 2014). Typically, these folding prediction methods estimate the free energy change of folding (∆∆G) due to mutation from 3D structures in addition to scores derived from different force-fields or evolutionary information. Encouragingly, about three-fourths of variants that cause Mendelian disorders affect protein stability (Wang and Moult 2001; Yue et al. 2014), suggesting that folding stability prediction methods can prioritize candidate disease drivers (Bocchini et al. 2016; Pey et al. 2007; Siekierska et al. 2012) for chaperone treatment (Chaudhuri and Paul 2006). However, these methods are partially limited by the availability of protein structure data. Although the Research Collaboratory for Structural Bioinformatics Protein Data Bank (RCSB PDB) currently contains more than 50,000 human protein structures, many are redundant and 30% of the human proteins have no PDB structure that corresponds to their sequence or any homologous sequence with as low as 30% sequence identity (Somody et al. 2017). Alternatively, stability prediction methods may take advantage of the recent development of high-quality protein structure predictors, such as AlphaFold (Jumper et al. 2021) and RosettaFold (Baek et al. 2021), which broaden the available number of protein structures. However, evolution-based stability prediction methods have competitive performance, despite the fact they do not use any structure information (Fariselli et al. 2015; Montanucci et al. 2019).

Toward broader genome interpretation of variant effect, other prediction methods rely on protein sequence homology primarily, supported when available by function annotations and structural information. Table 1 lists well-established representatives of these methods, grouped according to the type of input data they use and whether the input includes scores from available predicting methods (Ensemble). Homology-based methods hypothesize that the frequent substitutions across clades are benign, under the implied assumption that homologous proteins share identical functions. Inversely, substitutions that do not occur across phylogenetic branches indicate negative selection and possible pathogenicity (Ng and Henikoff 2001). To overcome limitations that stem from this hypothesis, homology-based prediction methods employ various techniques, such as pseudocounts (Henikoff and Henikoff 1996), phylogenetic distances (Davydov et al. 2010; Katsonis and Lichtarge 2014; Reva et al. 2011), Hidden Markov Models (Garber et al. 2009; Rogers et al. 2018; Shihab et al. 2013, 2015; Siepel et al. 2005; Thomas et al. 2003), similarity scores (Choi et al. 2012), normalized probabilities in localized sequence segments (Capriotti et al. 2006), and restricting sequence alignments to mostly orthologous proteins (Mathe et al. 2006). These homology-based prediction methods are different from methods of residue importance (see a selected list in Table 2), which provide a "conservation" score for each protein residue rather than for each amino acid substitution.

**Table 1:** Representative predictors of variant fitness effects

Type	Method name	Input features	Training dataset	Validating datasets	Paper	Citations
Homology	Align-GVGD	Multiple sequence alignment	–	Exp: p53 Clin: IARC p53	Tavtigian et al. (2006)	655
Homology	DeMaSk	Multiple sequence alignment	Amino acid substitutions from 18 deep mutational scanning studies	Leave one out	Munro and Singh (2020)	6
Homology	EFIN	Sequence conservation features	UniProt, HumDiv	SwissProt	Zeng et al. (2014)	25
Homology	Evolutionary Action	Multiple sequence alignment	–	Exp: lacI, lysozyme, HIV protease, RecA, p53 Clin: IARC p53, UniProt, CFTR, GAA Pop: 1000 Genomes	Katsonis and Lichtarge (2014)	105
Homology	FATHMM	Multiple sequence alignment	–	Exp: VariBench Clin: HGMD, UniProt, SwissVar Oth: Hicks et al., 2011 (BRCA1, MSH2, MLH1, TP53)	Shihab et al. (2013)	932
Homology	likelihood ratio test (LRT)	Multiple sequence alignment	–	SNPs of three individualexomes OMIM database	Chun and Fay (2009)	920
Homology	MAPP	Multiple sequence alignment Phylogenetic Tree	–	Exp: lacI, lysozyme, HIV protease, HIV RT Clin: pyruvate kinase, G6PD, HBB, IARC p53	Stone and Sidow (2005)	371
Homology	MutationAssessor	Multiple sequence alignment	–	UniProt, IARC p53, COSMIC	Reva et al. (2011)	1687
Homology	PANTHER	Multiple sequence alignment	–	Clin: HGMD, dbSNP, Pop: SNPs sampled from healthy individuals	Thomas et al. (2003)	2924
Homology	PhD-SNP	Multiple sequence alignment Local sequence environment	Swiss-Prot, HumVar	Newer HumVar variants	Capriotti et al. (2006)	732
Homology	PrimateAI	Sequence alignments and sequence-based predictions of structure features	Path: SNVs absent from ExAC Ben: common SNVs and primate changes	Withheld variants from training	Sundaram et al. (2018)	183
Homology	PROVEAN	Multiple sequence alignment	–	Exp: LacI, TP53, ABCA1 Clin: UniProtKB/Swiss-Prot	Choi et al. (2012)	2445
Homology	SIFT/SIFT4G	Multiple sequence alignment	–	Exp: lacI, lysozyme, HIV protease Clin: HumDiv, HumVar	Ng and Henikoff (2003)	5242
					Ng and Henikoff (2001)	2543
					Vaser et al. (2016)	691
Multiple features	DeepSAV	16 conservation, 28 structure and 1 dynamics score	ClinVar and UniProt	ClinVar and UniProt	Pei et al. (2020)	5
Multiple features	ENTPRISE	Alignment and structure related features	multiple sources, including: HGMD, UniProt, SwissProt	Clin: COSMIC, TCGA,COBR Pop: VariSNP, 1000 Genomes	Zhou et al. (2016)	22
Multiple features	Envision	homology, structure, amino acid properties, and more	large-scale experimental mutagenesis data	Withheld variants from training	Gray et al. (2018)	107
Multiple features	FATHMM-MKL	10 feature groups (conservation and function annotations)	Path: HGMD Ben: 1000 Genomes	ClinVar, newer HGMD variants	Shihab et al. (2015)	451
Multiple features	FATHMM-XF	31 feature groups (conservation, ENCODE, Epigenomics, and more)	Path: HGMD Ben: 1000 Genomes	ClinVar	Rogers et al. (2018)	174
Multiple features	MutationTaster/MutationTaster2	Conservation, sequence, and protein features	Path: HGMD, OMIM, and more Ben: 1000 Genomes, HapMap, and more	Withheld variants from training	Schwarz et al. (2010)	2675
					Schwarz et al. (2014)	2778
Multiple features	MutPred/MutPred2	Structure, function annotation, and evolutionary features	Path: HGMD, SwissProt, Cancer drivers Ben: SwissProt	Withheld variants from training/ClinVar, SwissVar, experimental data	Li et al. (2009)	773
					Pejaver et al. (2020)	190
Multiple features	PolyPhen/PolyPhen-2	Sequence and structure based features	HGVbase / Path: UniProt (HumDiv, HumVar) Ben: Differences between orthologs	HumVar	Ramensky et al. (2002)	2538
					Adzhubei et al. (2010)	11,660
Multiple features	PON-P2	Conservation, ontology functional, and structural	VariBench	Withheld variants from training	Niroula et al. (2015)	161
Multiple features	SNAP/SNAP2	Conservation, structure prediction, and more	Path: Protein Mutant Database Ben: Swiss-Prot	Exp: lacI, lysozyme, HIV protease, Melanocortin-4	Bromberg and Rost (2007)	807
					Hecht et al. (2015)	339
Multiple features	SNPs&GO	50 features of mutation, sequence profile, PANTHER output, and GO classification	Swiss-Prot	Disease classification from Goh et al. 2007	Calabrese et al. (2009)	600
Multiple features	SNPs3D	Structure related features/alignment related features	Path: HGMD Ben: Differences of homologous proteins	Clin: HGMD Pop: dbSNP	Yue et al. (2005)	452
					Yue and Moult (2006)	283
Multiple features	VEST	86 features of SNVBox	Path: HGMD Ben: ESP	Pop: SwissProt, 1000 Genomes	Carter et al. (2013)	342
Multiple features	VIPUR	20 protein sequence and structure-based features	UniProt	withheld variants from training	Baugh et al. (2016)	50
Functional genomic	fitCons	DNase-seq data, RNA-seq data, ChIP-seq data	–	Transcription factor binding sites expression quantitative trait loci (eQTL) enhancers based on characteristic chromatin marks	Gulko et al. (2015)	215
Ensemble +	CADD	63 features, including: predictors, conservation scores, and function annotation	Human-chimp changes simulated de novo variants	Exp: ALDOB, ECR11, HBB Clin: HGMD, MML2, ClinVar, IARC p53 Pop: ESP, GVS, 11 individuals	Kircher et al. (2014)	4681
Ensemble +	CAPICE	63 features, including: predictors, conservation scores, and function annotation	Path: ClinVar, VKGL Ben: ClinVar, VKGL, ExAC	ClinVar, VKGL and ExAC	Li et al. (2020)	9
Ensemble +	ClinPred	16 predictors and allele frequencies of gnomAD	ClinVar	Exp: BRCA1 Clin: mutagenetix database, DoCM,	Alirezaie et al. (2018)	79
Ensemble	Condel	5 predictors (Logre, MAPP, MutationAssessor, Polyphen2, and SIFT)	HumVar, HumDiv, COSMIC, p53	–	Gonzalez-Perez andLopez-Bigas (2011)	788
Ensemble +	DANN	949 features, including: predictors, conservation scores, and function annotation	Human-chimp changes simulated de novo variants	Clin: ClinVar Pop: ESP	Quang et al. (2015)	689
Ensemble +	DEOGEN / DEOGEN2	PROVEAN scores, alignment, Network, pathway, gene essentiality and more features	UniProt	Clin: UniProt, p53, F8, BRCA1	Raimondi et al. (2016)	26
					Raimondi et al. (2017)	61
Ensemble +	Eigen	Predictors, conservation scores and allele frequencies of the 1000 Genomes	ClinVar	De novo variants in several studies	Ionita-Laza et al. (2016)	408
Ensemble	InMeRF	28 predictors and 9 conservation scores	Path: HGMD Ben: variants with MAF > 0.1%	VariBench, PredictSNP, SwissVar	Takeda et al. (2020)	3
Ensemble +	M-CAP	9 predictors, 7 conservation scores, and 298 alignment-based features	Path: HGMD (AF < 1%) Ben: ExAC (AF < 1%)	Withheld variants from training	Jagadeesh et al. (2016)	531
Ensemble	MetaLR MetaSVM	15 predictors and 3 conservation scores	UniProt	VariBench, CHARGE database, and publications	Dong et al. (2015)	769
Ensemble	Meta-SNP	4 predictors: PANTHER, PhD-SNP, SIFT, and SNAP	SwissVar	Newer SwissVar variants	Capriotti et al. (2013)	176
Ensemble +	MISTIC	7 predictors, 8 conservation scores, MAF, and genetic and protein function	Path: ClinVar Ben: gnomAD	Clin: new ClinVar Pop: SweGen, UK10K, and more	Chennen et al. (2020)	12
Ensemble +	MPC	PolyPhen2 and other deleteriousness metrics	Path: ClinVar Ben: common ExAC variants	5620 neurodevelopmental disorder cases and 2078 controls	Samocha et al. (2017)	146
Ensemble +	MutScore	5 Predictors: SIFT, SIFT4G, LRT, PROVEAN, GERP + + RS and 9 conservantion scores	ClinVar	ClinVar	Quinodoz et al. (2022)	0
Ensemble +	MVP	15 predictors, 6 conservation scores, structure, interactions, gene intolerance, and more	Path: HGMD, UniProt, ClinVar Ben: UniProt, and more	Path: VariBench, Cancer hotspots Ben: DiscovEHR	Qi et al. (2021)	25
Ensemble	PON-P	5 predictors: PhD-SNP, SIFT, PolyPhen-2, SNAP, I-Mutant	Path: PhenCode, Idbases, and more Ben: dbSNP with AF > 0.1	Protein Mutant Database	Olatubosun et al. (2012)	108
Ensemble	PredictSNP/PredictSNP2	8 predictors / 6 predictors	UniProt and training datasets of: SNPs&GO, MutPred, and PON-P / ClinVar,GWAS catalog, COSMIC, VariSNP	Protein Mutant Database, and experimental studies/Mendelian disease and cancer driver variants	Bendl et al. (2014)	485
					Bendl et al. (2016)	119
Ensemble	REVEL	10 predictors and 8 conservation scores	Path: HGMD Ben: ESP, ARIC, 1000 Genomes	ClinVar and SwissVar	Ioannidis et al. (2016)	853
Ensemble +	Rhapsody	4 sequence, 1 structure, and 4 dynamic scores	HumVar, ExoVar, PredictSNP, VariBench, and SwissVar	HumVar, ExoVar, PredictSNP, VariBench, and SwissVar	Ponzoni et al. (2020)	32

Methods citations (Adzhubei et al. 2010; Alirezaie et al. 2018; Baugh et al. 2016; Bendl et al. 2014, 2016; Bromberg and Rost 2007; Calabrese et al. 2009; Capriotti et al. 2013, 2006; Carter et al. 2013; Chennen et al. 2020; Choi et al. 2012; Chun and Fay 2009; Dong et al. 2015; Gonzalez-Perez and Lopez-Bigas 2011; Gray et al. 2018; Gulko et al. 2015; Hecht et al. 2015; Ioannidis et al. 2016; Ionita-Laza et al. 2016; Jagadeesh et al. 2016; Katsonis and Lichtarge 2014; Kircher et al. 2014; Li et al. 2009, 2020; Munro and Singh 2020; Ng and Henikoff 2001, 2003; Niroula et al. 2015; Olatubosun et al. 2012; Pei et al. 2020; Pejaver et al. 2020; Ponzoni et al. 2020; Qi et al. 2021; Quang et al. 2015; Quinodoz et al. 2022; Raimondi et al. 2016, 2017; Ramensky et al. 2002; Reva et al. 2011; Rogers et al. 2018; Samocha et al. 2017; Schwarz et al. 2010; Shihab et al. 2013, 2014, 2015; Stone and Sidow 2005; Sundaram et al. 2018; Takeda et al. 2020; Tavtigian et al. 2006; Thomas et al. 2003; Vaser et al. 2016; Yue et al. 2005; Yue and Moult 2006; Zeng et al. 2014; Zhou et al. 2016)

*Method inclusion criteria:

(i) be applicable on missense mutations

(ii) provide a single value for the impact of mutations

(iii) the impact should represent the overall effect on the protein function

*Path *pathogenic, *Ben *benign, *Exp *experimental associations, *Clin* clinical associations, *Pop *population data

**Table 2:** Predictors of residue importance

Type	Method name	Input features	Validating datasets	Paper
Homology	ConSurf	Multiple sequence alignment	SH2 and PTB signaling domains/Bcl-XL/Bak peptide complex	Armon et al. (2001) Glaser et al. (2003)
Homology	Evolutionary Trace	Multiple sequence alignment	ligand binding sites (SH2, SH3 domains, DNA binding)/PDB structures with ligands bound	Lichtarge et al. (1996) Mihalek et al. (2004)
Homology	GERP + +	Multiple sequence alignment	PolII binding regions (ENCODE)	Davydov et al. (2010)
Homology	PhastCons	Multiple sequence alignment	–	Siepel et al. (2005)
Homology	PSIC	Multiple sequence alignment	–	Sunyaev et al. (1999)
Homology	Rate4Site	Multiple sequence alignment	Src SH2 domain	Pupko et al. (2002)
Homology	SiPhy	Multiple sequence alignment	ENCODE regions	Garber et al. (2009)
Ensemble	PhyloP	4 conservation scores: LRT, SCORE, SPH, GERP	ENCODE	Pollard et al. (2010)

Methods citations (Armon et al. 2001; Davydov et al. 2010; Garber et al. 2009; Glaser et al. 2003; Lichtarge et al. 1996; Mihalek et al. 2004; Pollard et al. 2010; Pupko et al. 2002; Siepel et al. 2005; Sunyaev et al. 1999)

To further improve variant effect predictions, additional insights into molecular functions (Calabrese et al. 2009) and physicochemical characteristics (Stone and Sidow 2005) can be considered, posing the problem of how to weigh the contributions of heterogeneous information sources. For this reason, machine learning techniques are routinely used to select and combine the numerous features that may indicate pathogenic or benign effects. Developers may select different techniques that work best for their purpose, including Support Vector Machines (Kircher et al. 2014; Yue et al. 2006), Random Forests and other Decision Trees (Carter et al. 2013; Raimondi et al. 2016, 2017; Ramensky et al. 2002; Zhou et al. 2018a), Neural Networks (Hecht et al. 2015; Qi et al. 2021; Quang et al. 2015; Sundaram et al. 2018), Naïve Bayes (Adzhubei et al. 2010), Logistic Regression (Baugh et al. 2016), or combinations of these (Li et al. 2009).

**Fig. 1:**
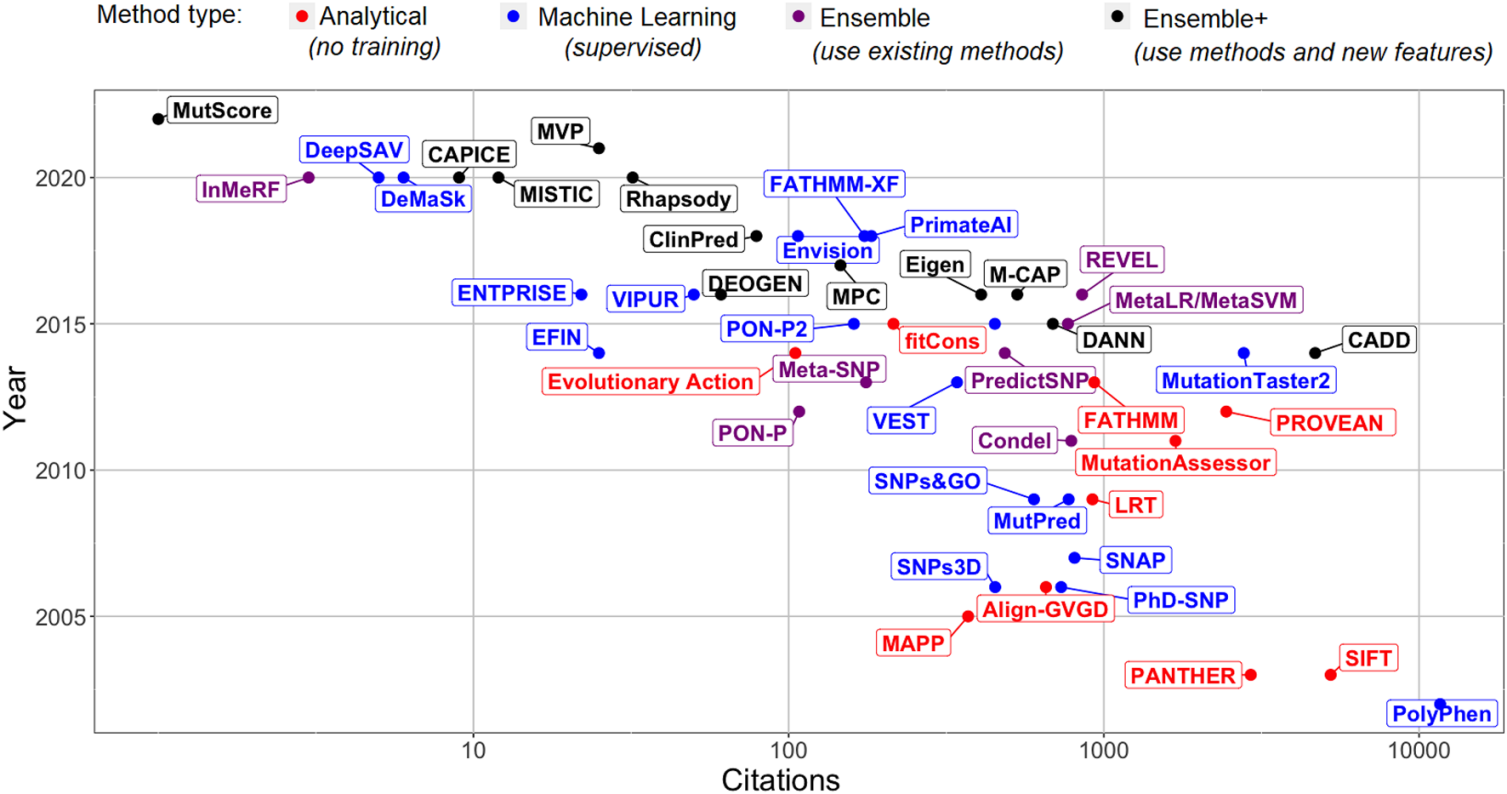
Number of citations to the primary paper of variant prediction methods as a function of the year it was published. The number of citations were obtained by Google Scholar search on the 7th of March 2022. When methods could be matched to multiple primary papers or newer versions were introduced, the paper with the most citations was used here. Methods are classified as (i) analytical models not trained on available variant annotations (red color), (ii) machine learning approaches trained on variant annotations (blue color), (iii) ensemble models that integrate scores from available predictors (purple color), and (iv) models that combine scores from available predictors and additional features (black color)

Some methods pool pre-existing prediction methods to estimate the protein function effects of variants with better accuracy. In Table 1, we labeled as "*Ensemble*" the meta-methods that combine multiple pre-existing methods to obtain a single overall protein function impact score. Machine-learning approaches are typically used for such purposes. However, some of these methods have used pre-existing prediction method scores together with additional features in their training, so we labeled them as "*Ensemble* + ". The method DANN (Quang et al. 2015) used the same feature set and training data as the method CADD (Kircher et al. 2014), but a different learning approach, and we subsequently labeled it "*Ensemble* + ". Figure 1 reflects the popularity for a large collection of available methods, indicated by the citations of the original articles as a function of the publication year.

### The aim of this review

In view of this diversity and importance of variant impact prediction methods, several reviews discuss the most common tools for predicting pathogenic variations, focusing on their underlying principles (Cline and Karchin 2011; Hassan et al. 2019; Katsonis et al. 2014; Tang and Thomas 2016), their clinical value (Ghosh et al. 2017; Yazar and Ozbek 2021; Zhou et al. 2018b), their mutual agreement (Castellana and Mazza 2013), or the integration of large-scale data (Cardoso et al. 2015; Chakravorty and Hegde 2018). Here, this review assesses the agreement of variant effect prediction methods with experimental and clinical data, summarizes their performance in objective blind challenges, and presents some of their successful applications to interpret variant impact and link genes to phenotypes. We find that, despite common concerns regarding performance (Castellana and Mazza 2013; Flanagan et al. 2010; Mahmood et al. 2017), numerous practical applications show that variant effect prediction methods prove reliable when used cautiously in the proper context. Thus, objective assessment exercises (Andreoletti et al. 2019; Hoskins et al. 2017) are critical to define appropriate use cases for each method, to set expectations for accuracy, and to evaluate performance improvements due to methodological refinements. Together with the interpretation of other variant types (Liu et al. 2019; Spurdle et al. 2008; Zeng and Bromberg 2019) and the integration with multi-omics data (Gomez-Cabrero et al. 2014; Huang et al. 2017a; Subramanian et al. 2020), variant effect prediction methods can help in understanding the genotype–phenotype relationship.

## Main text

### Method training and validation

Most methods estimate variant impact by weighing homology and/or structural information in unique ways. Homology-based predictors use multiple sequence alignments (MSA) to achieve their goal, which may either be narrow sets of orthologous sequences or larger sets that include distant homologs and paralogs. The choice of MSA affects the performance of the prediction methods and using the MSA provided by each method does not guarantee optimal performance (Hicks et al. 2011). MSA are also useful in predictors that go beyond basic homology and infer additional properties. Distinct properties are captured by conservation scores (Sunyaev et al. 1999), phylogenetic correlations of residues (Lichtarge et al. 1996), amino acid substitution frequency (Henikoff and Henikoff 1992), biochemical properties at the position of interest (Grantham 1974) and whole substitution profiles. These properties may define the context in which the mutation is observed, together with protein structure variables when a protein structure is available. Solvent accessibility (Ahmad et al. 2004; Lee and Richards 1971), secondary structure (Kabsch and Sander 1983), B-factors (Sun et al. 2019), intrinsic disorder (Dunker et al. 2002), and ligand binding sites (Hendlich et al. 1997; Yang et al. 2013) provide insights into changes occurring in the protein that can only be minimally inferred by homology-based methods. Given the plethora of features to choose and combine, differences between variant impact predictors are not surprising. In addition, many methods make use of various training data to refine predictions and select the most important features. The type of data chosen to train a method will likely influence the predicted impact to be more relevant to experimental, clinical, or evolutionary effects.

### Experimental data to train and validate prediction methods

To validate, benchmark, and compare methods, predictors may turn to the experimental outcomes of large mutagenesis studies. For this purpose, four readily accessible datasets gained special popularity and were frequently used: (i) the repression activity of 4041 lac repressor mutations in E. coli (Markiewicz et al. 1994), (ii) the transactivation activity of 2314 human p53 mutations (Kato et al. 2003), (iii) the break-up of the host cell walls due to 2015 lysozyme mutations in bacteriophage T4 (Rennell et al. 1991), and (iv) the cleavage of Gag and Gag-Pol due to 336 HIV-1 protease mutations (Loeb et al. 1989). More broadly, some dedicated databases curate experimental variation data for benchmarking (Kawabata et al. 1999; Sasidharan Nair and Vihinen 2013). In addition, many more large mutagenesis studies are now available and could be used for additional benchmarking (Gray et al. 2017; Livesey and Marsh 2020; Sruthi et al. 2020). Overall, variant impact prediction methods using these large datasets perform well, if not superbly, according to developer benchmark analyses. Pearson’s correlations were nearly perfect when the experimental data were binned (Katsonis and Lichtarge 2014), showing excellent agreement with the overall trend despite point-by-point fluctuations. Accuracy was about 70% for SNAP (Bromberg and Rost 2007), 68–80% for SIFT (Ng and Henikoff 2001), and slightly better for MAPP (Stone and Sidow 2005). Areas under the ROC curve (AUC) have been reported to be 81–87% for PROVEAN (Choi et al. 2012) and from 86 to 89% for Evolutionary Action (Katsonis and Lichtarge 2014).

### Clinical data to train and validate prediction methods

For further training and validation, prediction methods can also call upon databases of clinical annotations. A well-known source of clinical associations is the ClinVar repository which reports associations between genetic variation and clinical phenotypes (Landrum et al. 2018). Variants in this database were found in patient samples and annotated according to the guidelines of the American College of Medical Genetics (ACMG) and of the Association for Molecular Pathology (AMP) (Richards et al. 2015), with the goal to provide clinicians with the most robust consensus assessment. The ClinGen group convened Variant Curation Expert Panels to validate genetic annotations for specific genes or sets of genes and update variant annotations in ClinVar (Rehm et al. 2015; Rivera-Munoz et al. 2018). An alternative dataset that is frequently used in training variant impact predicting methods is the Human Gene Mutation Database (HGMD) (Stenson et al. 2020). HGMD is a manually curated, proprietary collection of published germline mutations in nuclear genes associated with human inherited disease. Also, HumSavar is an open access collection of missense variant reports, curated from literature according to ACMG/AMP guidelines (Richards et al. 2015), and it is available through UniProtKB/Swiss-Prot (Wu et al. 2006). Moreover, Cancer-specific sources are available for somatic and germline variants, such as COSMIC (Tate et al. 2019), International Agency for Research on Cancer (IARC) (Petitjean et al. 2007), Database of Curated Mutations (DoCM) (Ainscough et al. 2016), CanProVar (Li et al. 2010), and BRCA Exchange (Cline et al. 2018). Other sources include LOVD (Fokkema et al. 2005), HuVarBase (Ganesan et al. 2019), and disease-specific collections, such as Pompe disease (Kroos et al. 2008), Wilson disease (Kumar et al. 2020), and others (Gout et al. 2007; Peltomaki and Vasen 2004). The mouse-specific database Mutagenetix (Wang et al. 2018, 2015) also contains genotype–phenotype correlations, using various phenotype assays. Using these databases, many studies showed that the disease driver variants are strongly enriched for pathogenic predictions. Performance, however, varies with the choice of reference dataset and the confidence level of the clinical association, and typically ranges between AUCs of 70% and 90% (Choi et al. 2012; Dong et al. 2015; Ioannidis et al. 2016; Niroula et al. 2015; Pejaver et al. 2020; Qi et al. 2021). Nearly perfect AUCs are occasionally reported (Alirezaie et al. 2018; Ghosh et al. 2017), but such outstanding performances may be overly optimistic and prone to indirect circularity (Grimm et al. 2015).

### Population fitness effect data to train and validate prediction methods

Population-wide sequencing efforts are also used to train or validate predictions. The pioneering 1000 Genomes Project ran from 2008 to 2015 and sequenced more than 2500 individuals across 26 populations from five continental groups (The 1000 Genomes Project Consortium 2015). Since 2015, the International Genome Sample Resource (IGSR) maintains The 1000 Genomes Project (Clarke et al. 2017) and added, thus far, samples from sources such as The Gambian Genome Variation Project (Malaria Genomic Epidemiology Network 2019), The Simons Genome Diversity Project (Mallick et al. 2016), and The Human Genome Diversity Project (Bergstrom et al. 2020). Larger population-wide sequencing datasets, such as the Exome Aggregation Consortium (ExAC) (Karczewski et al. 2017; Lek et al. 2016) and the more recent Genome Aggregation Database (gnomAD) (Karczewski et al. 2020), seek to aggregate variants from various projects. These projects mostly include data from disease-specific studies. ExAC and gnomAD provide allele frequency information instead of the individuals’ genomes and may contain more disease driver variants than expected in healthy individuals. Other databases describing population-wide variation include the UK Biobank (Sudlow et al. 2015), dbSNP (Markiewicz et al. 1975), the Exome Variant Server (NHLBI Exome Sequencing Project 2011), HapMap (The International HapMap Consortium 2003), and various population-specific data sets (All of Us Research Program et al. 2019; Ameur et al. 2017; GenomeAsia 100K Consortium 2019; Jain et al. 2021; John et al. 2018; Jung et al. 2020). These sources of human polymorphisms often provide the benign set of variants for training many predictors (such as FATHMM –MKL and –XF, M-CAP, MISTIC, MPC, MutationTaster, PON-P, PrimateAI, REVEL, and VEST). The underlying hypothesis is that human polymorphisms have been under negative selection pressure that may eliminate or prevent the spread of many pathogenic variants. Therefore, most of the observed variants, especially those with high allele frequency should have nearly neutral or positive effects on protein function (Kimura 1979). Prediction scores that were not trained on human polymorphisms supported this hypothesis, showing that human polymorphisms were enriched in low pathogenicity scores, with the enrichment becoming stronger for variants with higher minor allele frequency (Katsonis and Lichtarge 2014). Another study used VEST to show that sources based on complete genomes, such as The 1000 Genomes Project, contain less pathogenic-predicted variants than sources of compiled clinical data, such as SwissProt (Carter et al. 2013). However, pathogenic variants exist within clinical genomes and studies suggest that predictors of protein function effects can prioritize them to identify candidate disease variants (Chennen et al. 2020; Ioannidis et al. 2016; Jagadeesh et al. 2016).

### Performance assessment of variant impact prediction methods – CAGI challenges

The performance of variant impact prediction methods is hard to assess unambiguously. Independent studies (Chan et al. 2007; Dong et al. 2015; Ghosh et al. 2017; Gunning et al. 2020; Leong et al. 2015; Li et al. 2018; Livesey and Marsh 2020; Michels et al. 2019; Miosge et al. 2015; Suybeng et al. 2020; Tian et al. 2019; Yadegari and Majidzadeh 2019) have compared a limited number of methods each, using specific sets of variants and evaluation tests, but ignoring potential training circularities, and making cutoff assumptions that may not fit each method equally well. In contrast, efforts to systematically and objectively assess variant impact prediction methods come from the Critical Assessment of Genome Interpretation (CAGI) community. CAGI, so far, has organized five assessment experiments that include many different challenges, with a new, sixth assessment currently in progress. The challenges may evaluate prediction performance with respect to the impact of variants on a specific protein function (Clark et al. 2019; Kasak et al. 2019a; Xu et al. 2017), clinical sequalae (Carraro et al. 2017; Cline et al. 2019; Voskanian et al. 2019), or global fitness, such as in yeast competition assays (Zhang et al. 2019, 2017a). Critically, all CAGI challenges use new and unpublished data, developer groups make predictions blind to pathogenic associations, and independent judges use multiple criteria to score success blind to the developer identity. The aims of these challenges are to recognize advantageous strategies used by the developers and bottlenecks that prevent the field from advancing. This goal is achieved through direct comparison of each method’s performance and the features they use. In our view, the performance on the CAGI challenges did not point to obvious links between the type of predictor and the type of challenge, because it was subject to several cofounding factors (including input data availability, participation, predictor adjustments and approximations, assessor choices, assay or clinical data interpretation). Some methods clearly performed better than others according to multiple assessment metrics, but often different metrics indicated different top methods for the same challenge, highlighting the need for combining multiple metrics. Consistently top-ranked predictions come from the Evolutionary Action (Katsonis and Lichtarge 2017, 2019), MutPred (Pejaver et al. 2017), SNAP (Kasak et al. 2019a), and ensemble methods (Yin et al. 2017). It is worth noting that many less well-known participating methods showed better performance than PolyPhen2 and SIFT, which are very popular and widely used variant impact prediction methods (Katsonis and Lichtarge 2017). Very simple predictive models, such as baseline sequence conservation predictors, may perform on par or better than sophisticated methods (Zhang et al. 2019, 2017a). Also, for a given approach (submissions from the Yang & Zhou lab on the cell proliferation rates upon CDKN2A variants), gradually adding features to the prediction model led to gradual performance improvements (Carraro et al. 2017), indicating that future development should focus on the aggregation, refinement, and validation of new features. In the PCM1 challenge that evaluated 38 human missense mutations implicated in schizophrenia with a zebrafish model assay (Monzon et al. 2019), all submitted predictions had poor performance and yielded a nearly random distribution of balanced accuracy (Katsonis and Lichtarge 2019), leaving questions about the source of disagreement (Miller et al. 2019). In the CALM1 gene challenge, the performance of all submitted predictions improved when the yeast complementation assay data points were limited to those with gradually smaller experimental standard deviation (Katsonis and Lichtarge 2019), indicating a potential underestimation of the performance in CAGI challenges due to experimental errors.

In addition to these variant-level challenges, exome-level CAGI challenges have suggested that variant effect prediction methods can help in finding the disease risk of individuals (Cai et al. 2017; Chandonia et al. 2017; Daneshjou et al. 2017). However, addressing such challenges requires weighing the contribution of genes to each trait and combining the effects of multiple variants, which are not straightforward, so the successes were limited. Three CAGI challenges were related to predicting the risk of Crohn`s disease, with two of them resulting in unreliable performance evaluations due to sample stratification issues and the third one showing AUCs of up to 0.7 (Giollo et al. 2017). The performance was slightly worse for predicting the risk for venous thromboembolism, with AUCs up to 0.65 and accuracies up to 0.63 (McInnes et al. 2019). In a more complex challenge, two methods performed significantly better than chance for matching the clinical descriptions of undiagnosed patients (Kasak et al. 2019b). However, it proved harder to distinguish between individuals of different intellectual disability phenotypes (Carraro et al. 2019). Overall, predicting the risk of individuals for complex diseases remains challenging and methods predicting variant effect may complement current efforts to solve the etiology of more cases.

### Applications of variant impact prediction methods

The methods predicting variant impact are already used in numerous practical applications, as indicated by the thousands of citations of PolyPhen2 (Adzhubei et al. 2010), SIFT (Kumar et al. 2009), and CADD (Kircher et al. 2014), amongst other methods. Typically, they are used to assess the impact of new variants of unknown significance, narrow down driver candidates, and to support evidence for pathogenic effects. The ACMG and AMP guidelines encourage using multiple lines of computational evidence to support pathogenic or benign classification (Richards et al. 2015). In addition, variant impact prediction methods have been used to guide mutagenesis studies and associate genes to phenotype. Next, we discuss representative practical applications of predicting methods that illustrate their value in genome interpretation. Although these applications regard specific predicting methods each, the CAGI experiments suggest that other methods could be equally or more successful in addressing the same scientific questions.

### Guiding mutagenesis studies

Targeted mutagenesis experimental studies can take advantage of the variant impact predicting methods to reduce experimental cost and effort while maximizing return without missing key results (Sruthi and Prakash 2020). However, such applications are rare and typically complement other prioritization strategies that account for the available protein structures and methods that predict protein residue importance, such as the methods in Table 2. For example, evolutionarily important residues were used to identify functional motifs in the DNA-dependent protein kinase catalytic subunit and the analysis of variant impact unveiled functional insights and implications (Lees-Miller et al. 2021). Variant effect prediction methods indicated human NAGK polymorphisms that reduced its binding to the dynein subunit DYNLRB1, an interaction that promotes cellular growth and other functions (Dash et al. 2021). Mutation impact and important residues also guided mutagenesis studies aiming to uncover the interaction between the RecA and LexA protein in E. coli, which controls antibiotic resistance (Adikesavan et al. 2011; Marciano et al. 2014). In G-protein-coupled receptors, variant impact scores correlated with the phenotypic change (Gallion et al. 2017; Schonegge et al. 2017), and they were used in selecting targeted mutations that recode the allosteric pathway specificity (Peterson et al. 2015; Rodriguez et al. 2010; Schonegge et al. 2017). More recently, variant impact score analysis guided the development of a mutant esterase that gained stereospecificity properties while maintaining a 53-substrate repertoire (Cea-Rama et al. 2021). These examples show that variant effect prediction methods can effectively prioritize and reduce the workload of mutagenic experimental studies.

### Supporting clinical associations

Methods predicting variant impact can help with the association of variants to traits, which currently relies on observational statistics in family studies (Borecki and Province 2008) and genome-wide association studies (GWAS) (Bush and Moore 2012; MacArthur et al. 2017). Because of linkage disequilibrium (Pritchard and Przeworski 2001), GWAS variants do not necessarily indicate causal effects (Cooper and Shendure 2011), highlighting the need for a transition to functional associations (Gallagher and Chen-Plotkin 2018). Currently, predicting methods are used in both monogenic and polygenic traits, supporting pathogenic effects for variants observed in cases and showing that candidate disease drivers are enriched in pathogenic scores, collectively. Any predicting method or the consensus of multiple methods can serve this supporting role. Few examples are variants causing FARS2 deficiency (Almannai et al. 2018), ANO5 variants causing Gnathodiaphyseal Dysplasia (Otaify et al. 2018), DHCR24 variants causing Desmosterolosis (Schaaf et al. 2011), variants in multiple genes causing ALS (Gibson et al. 2017; Kenna et al. 2016), Parkinson's disease (Oluwole et al. 2020), and Alzheimer’s disease (Vardarajan et al. 2015). Similar analyses for somatic mutations in tumors revealed strong selection patterns, either on numerous candidate genes (Bailey et al. 2018) or on specific genes such as in PTPN12 (Nair et al. 2018), BAP1 (Sharma et al. 2019), and TP53 (Li et al. 2016). Therefore, computational approaches can prioritize somatic variants for their role in cancer, using variant effect predictors and gene features (Kaminker et al. 2007). Predicting methods may also prioritize germline variants of trait-associated genes for further examination, such as in cardiovascular diseases (Rababa'h et al. 2013; Suryavanshi et al. 2018; Wang et al. 2021b). With the spread of SARS-CoV-2, computational prediction methods have presented a functional site overview for all SARS-CoV-2 proteins (Wang et al. 2021a) and suggested that their mutational hotspots can alter protein stability and binding affinity (Teng et al. 2021; Wu et al. 2021; Zou et al. 2020). These studies show that predicting methods have practical value in a variety of clinical associations, in both Mendelian and complex diseases, including cancer.

### Informing diagnoses and clinical decision making

Variant impact predictors may extend from the bench to the bedside. First tier clinical tests typically use chromosomal microarrays, with reported diagnostic yield of 15–20% in patients with developmental disabilities or congenital anomalies (Miller et al. 2010). Unexplained cases may proceed to whole exome sequencing (WES) or whole genome sequencing (WGS) to fill this gap. Variant impact predictors can aid in the interpretation of the sequenced variants, offering significant increase in diagnostic yield (Grunseich et al. 2021; Stavropoulos et al. 2016). Pharmacogenomics studies may take advantage of predicting methods to interpret the impact of amino acid variations on drug metabolism (Isvoran et al. 2017; Matimba et al. 2009). Additionally, the predicted pathogenicity of somatic mutations in cancer was used in a classification system that may inform patient management (Sukhai et al. 2016). In a study of how TP53 variants affect the health of head and neck cancer patients, Evolutionary Action was able to stratify the overall survival and time to metastasis (Neskey et al. 2015), indicated resistance to cisplatin therapy (Osman et al. 2015b), and provoked suggestions for personalized treatment (Osman et al. 2015a). Similarly, survival stratification was obtained in two independent studies for colorectal liver metastases patients (Chun et al. 2019) and myelodysplastic syndrome patients (Kanagal-Shamanna et al. 2021). Therefore, predicting methods contribute in clinical diagnosis and can open paths toward precision medicine.

### Associating genes to phenotype

Variant impact scores may lead to associations of genes to traits. Typically, gene-trait associations rely on detecting selection patterns within a group of individuals who share the trait (cases) compared to unaffected individuals (controls). These selection patterns arise because trait driver genes harbor several pathogenic variants in cases, in addition to non-pathogenic variants that may appear in either the cases or the controls. Current gene discovery methods may quantify patterns such as whether the gene has more mutations compared to the expected number (Lawrence et al. 2013), the mutations cluster in the protein structure or sequence compared to homogeneous spread (Tamborero et al. 2013), and the characteristic nucleotide context of the mutations differs from the context of all other mutations (Dietlein et al. 2020). Since the driver variants have larger predicted pathogenicity values compared to random nucleotide substitutions, methods that predict protein function effects offer an additional pattern toward pointing to candidate trait-driver genes. This selection pattern is orthogonal and complementary to the aforementioned measures, making variant impact prediction methods valuable for gene discovery. Next, we note such applications to somatic, de novo, and inherited variants.

### Genes under selection in somatic mutations

Many cancer studies use variant impact prediction methods either as supporting evidence for the pathogenicity of gene variants (Bailey et al. 2018; Cancer Genome Atlas Research Network 2011) or as the main evidence to establish a gene-cancer link through an automated discovery process (Davoli et al. 2013; Gonzalez-Perez and Lopez-Bigas 2012; Hsu et al. 2022; Parvandeh et al. 2022). The underlying hypotheses are that most somatic mutations are passengers (i.e. they do not contribute to oncogenesis) and that driver mutations (i.e. they contribute to the development of cancer) occur selectively in specific genes (Greenman et al. 2007; Stratton et al. 2009). Because the driver variants affect protein function, predicting methods should statistically score driver variants as more pathogenic than passenger variants (Carter et al. 2009; Chen et al. 2020; Cline et al. 2019; Mullany et al. 2015; Reva et al. 2011) and point to cancer driver genes. Moreover, protein effect prediction methods can inform regarding the role of each gene in cancer, with tumor suppressor genes having mostly loss-of-function variants with high impact scores and oncogenes having mostly gain-of-function variants with intermediate to high scores (Hsu et al. 2022; Shi and Moult 2011). Gene pathway information may complement variant impact prediction methods in finding cancer driver genes (Cancer Genome Atlas Research Network 2017), even for small patient sets, such as 29 patients with sporadic Parathyroid Cancer (Clarke et al. 2019). These applications suggest that variant impact prediction methods can help in finding candidate driver genes within whole cancer cohorts and within their cancer type divisions.

### Genes under selection in de novo mutations

Variant impact prediction methods are commonly used in prioritizing the functional effects of de novo variants (Hu et al. 2016; Pejaver et al. 2020; Wang et al. 2019; Willsey et al. 2017). However, de novo variants are typically absent from the general population, with each individual harboring less than two coding de novo variants (Iossifov et al. 2012; Sevim Bayrak et al. 2020). This fact limits the gene-level analysis of de novo variants, even for large datasets, such as the Simons Simplex Collection (SSC) (Fischbach and Lord 2010), which contains sequencing data from more than 2500 families with at least one child diagnosed with autism spectrum disorder (ASD) (Iossifov et al. 2012; Lord et al. 2020, 2018). Typically, such data are analyzed in the contexts of known gene-phenotype associations and the human interactome network (Chen et al. 2018). Variant impact prediction methods, such as MutPred2 (Pejaver et al. 2020) and VIPUR (Buja et al. 2018), have shown that de novo variants in ASD cases have a higher fraction of predicted pathogenic variants compared to healthy siblings. Going one step further, a study using the Evolutionary Action (EA) method and gene pathway information without prior knowledge of phenotype associations identified 398 genes (representing 23 pathways) as candidate drivers for ASD, based on the enrichment of de novo variants to pathogenic scores (Koire et al. 2021). The same study proposed polygenic risk scores based on the EA scores of either de novo or rare inherited variants on candidate genes and showed that these scores correlated with the Intelligence Quotient (IQ) of patients. These correlations were stronger when the contribution of each gene was weighted by Residual Variation Intolerance Scores (RVIS), a measure of genic intolerance to mutations (Petrovski et al. 2013). Similar analyses can be done for more phenotypes, such as congenital heart disease (Jin et al. 2017), where cases appear to have a higher fraction of predicted pathogenic variants compared to healthy controls (Qi et al. 2021). Such large family datasets provide de novo mutations that can use variant impact predictions together with other information to discover new genes toward decoding the genotype–phenotype relationship.

### Genes under selection in inherited mutations

Case–control studies are routinely designed for the discovery of genes associated with a particular trait. For Mendelian traits, these associations are straightforward, and methods predicting protein function effects can help (Carter et al. 2013; Hu et al. 2013). For complex traits, the standard is gene-based GWAS for the trait of interest using all variants within a gene rather than each variant individually (Huang et al. 2011; Liu et al. 2010), but phenome-wide association studies can also serve the same purpose (Denny et al. 2010). However, spurious associations resulting from correlations with the true risk factors can lead to false-positive results (Risch 2000). Mendelian randomization may be used to overcome confounding (Grover et al. 2017) and complementary analyses including, but not limited to, literature text-mining (Bhasuran and Natarajan 2018; Zhou and Fu 2018) and gene co-expression analyses (van Dam et al. 2018) can also help. Additionally, variant impact predictors can aid in deprioritizing variants predicted to have low functional impact, thus reducing such false positive discoveries (Lee et al. 2014; Wei et al. 2011). For example, FATHMM-XF, SIFT, PolyPhen2, and CADD were used to prioritize 190 candidate genes for driving neuroticism (Belonogova et al. 2021) and similarly for other traits (Bacchelli et al. 2016; Zhang et al. 2018). In CAGI challenges, many participants predicted the risk of individuals based on genomic data and matched genotypes to phenotypes better than random (Kasak et al. 2019b; Katsonis and Lichtarge 2019; Pal et al. 2017, 2020; Wang and Bromberg 2019). The imputed Deviation in Evolutionary Action Load (iDEAL) approach used protein function predictions to discover trait drivers (Kim et al. 2021). Specifically, it was applied to late-onset Alzheimer's disease (AD) patients that paradoxically carried the AD-protective APOE ɛ2 allele compared to healthy individuals that carried the AD-risk APOE ɛ4 allele. This study identified 216 genes with differential Evolutionary Action load between the two populations. These genes showed a robust predictive power even in the independent set of APOE ɛ3 homozygote individuals and are potential drug targets. Therefore, there is strong evidence that methods predicting protein function effects have the potential to help in genome interpretation of complex diseases in a post-GWAS era.

## Discussion

This review of current computational estimates of protein function effects due to variants illustrates several practical applications. They routinely guide experimental studies of protein structure and function and clinical studies of variants of unknown significance that are candidate disease drivers. Most recently, they played a major role in identifying new genes associated with traits, for either somatic, de novo, or inherited variants. This ability to translate genomic data into quantitative traits raises hope for improved diagnostic tests with polygenic risk scores that account for functional effects rather than relying only on observational statistics. A caveat is that the basis for most methods remains rooted in homology information. The scores will thus tend to assess long-term "evolutionary" effects. Generally, and depending on the prediction method and the test data, these effects will tend to align with clinical or experimental impact as shown by strong correlations through extensive validation studies and objective assessments. In other words, the fitness landscape may appear similar at different scales.

### Criticism and value

In the past, variant impact prediction methods sustained pointed criticism (Flanagan et al. 2010; Mahmood et al. 2017; Tchernitchko et al. 2004) and this curtailed their use as prognostic tools. Most often, the criticism was fed on the one hand by a demand for nearly perfect accuracy in clinical diagnostics (Walters-Sen et al. 2015), and on the other hand by disagreements, first, between different methods (Chun and Fay 2009), and second, between prediction methods and experimental data or clinical annotations (Mahmood et al. 2017; Miller et al. 2019). At some degree, these discrepancies are due to misalignments between the hypotheses adopted by the method developers and the data analysts: a key is useful only when it is properly applied to the right lock. In the light of epistatic interactions, inaccuracy is expected for single variant estimations, since each individual has a unique genetic, epigenetic, and environmental background that may modify the impact of this variant. These factors may result in incomplete penetrance, where two individuals with the same genetic variant can have either benign or disease phenotype linked to that variant (Cooper et al. 2013; Waalen and Beutler 2009; Zlotogora 2003). Predictors can explicitly capture residue dependencies between positions to improve accuracy (Hopf et al. 2017) and focused methods can detect covariation signals in multiple sequence alignments to identify residue pairs with epistatic effects (Jones et al. 2012; Morcos et al. 2011; Salinas and Ranganathan 2018; Shen and Li 2016). However, most predictors of protein function effects provide estimates in a broader view, as when individual background effects are averaged out over cohorts of individuals, suggesting they are more informative in high-penetrance genes and disorders. Literally, homology-based prediction methods ignore the context and answer whether a specific variant is pathogenic in an "*evolutionary sense*," which at best matches the human population at large rather than addressing the context-dependent effects of the variants (DiGiammarino et al. 2002). The choice of a multiple sequence alignment input defines the “*average context*” of the computation and its potential biases and errors will affect the accuracy of the predictions (Hicks et al. 2011). Each algorithmic approach weighs input features differently from the other methods, which may influence prediction accuracy dramatically. Since both the sequence alignment input and the algorithmic approach affect prediction accuracy, we should avoid generalizing the performance conclusions based on a single analysis. Moreover, the assessors should ensure their hypothesis does not conflict those underlying each prediction method. The CAGI challenges offer useful insights into the performance of different methods since method developers are able to modify their approach according to the needs of each challenge and independent assessors ensure objectivity. These assessments demonstrate progress in the field of variant impact prediction and the need to adjust predictors given specific tasks. Newer approaches achieve strong correlations with experimental assay data and perform consistently better than well-known methods (Katsonis and Lichtarge 2017). Such correlations may improve when the impact of experimental noise is reduced, using only data points with small standard deviations (Katsonis and Lichtarge 2019) or combining multiple experimental assays (Gallion et al. 2017). This suggests that even systematic assessments may under-estimate the performance of predicting methods.

### Predicting the impact of other variant types

Whole genome sequencing shows that non-synonymous variants are less than 0.3% of the total calls (Shen et al. 2013). There is therefore growing interest in prediction methods of other variant types. Stop-gain and frameshift insertion and deletion (fs-indel) variants result in protein sequence truncation and are traditionally viewed as pathogenic, but many of them appear frequently in human genomes even in a homozygous state (MacArthur and Tyler-Smith 2010). Non-frameshifting insertion and deletion (indel) variants are also of interest due to their link to diverse clinical effects and their substantial genetic load in most humans (Mullaney et al. 2010). Methods such as SIFT Indel (Hu and Ng 2012), DDIG-in (Folkman et al. 2015), VEST‐Indel (Douville et al. 2016), and MutPred-LOF/-Indel (Pagel et al. 2017, 2019) may use homology, structure, intrinsic disorder predictions, and gene importance features to prioritize nonsense and indel variants with reported balanced accuracy of 80–90% (Douville et al. 2016). PROVEAN (Choi et al. 2012) and MutationTaster2 (Schwarz et al. 2014) also provide predictions to non-frameshifting indel variants following the same framework they used for predicting the impact of missense variants. CADD (Kircher et al. 2014) is designed to predict the impact of all classes of genetic variation, including splice sites (Rentzsch et al. 2021) and non-coding variations. Methods that focus on predicting splicing effects use as input the genomic sequence of the pre-mRNA transcripts and include SpliceAI (Jaganathan et al. 2019), MutPred Splice (Mort et al. 2014), Human Splicing Finder (Desmet et al. 2009), SPiCE (Leman et al. 2018), and Skippy (Woolfe et al. 2010). Methods that focus on predicting noncoding variant effects rely on functional genomics data, such as various sequence conservation and constraint scores (Dousse et al. 2016; Garber et al. 2009; Siepel et al. 2005), in silico predictions of transcription factor binding sites, enhancer regions, and long noncoding RNAs (lncRNAs) (Abugessaisa et al. 2021; Fu et al. 2014; Loots and Ovcharenko 2004; Pachkov et al. 2013), and experimental evidence provided by the Encyclopedia of DNA Elements (ENCODE) (Davis et al. 2018; Encode Project Consortium 2012), including transcription factor ChIP-seq, DNA methylation arrays, and small RNA-seq projects. Some non-coding functional impact predictors include, but are not limited to, LINSIGHT (Huang et al. 2017b), GenoCanyon (Lu et al. 2015), FATHMM-MKL (Shihab et al. 2015), FATHMM-XF (Rogers et al. 2018), PAFA (Zhou and Zhao 2018), DIVAN (Chen et al. 2016), and GWAVA (Ritchie et al. 2014). Synonymous variants, despite often assumed to be benign, are implicated in many diseases (Zeng and Bromberg 2019). SiVA (Buske et al. 2013), TraP (Gelfman et al. 2017), DDIG-SN (Livingstone et al. 2017), regSNPs-splicing (Zhang et al. 2017b), IDSV (Shi et al. 2019), and synVep (Zeng et al. 2021) have used conservation, RNA, DNA, splicing, and protein features to prioritize synonymous variants with typical performances of 0.85–0.90 AUC (Zeng and Bromberg 2019). Although we still need to objectively assess these methods, they may be useful for a transition to whole-genome interpretation.

### Significance in personalized therapy

Genome interpretation relies on the classification of genetic variants as pathogenic or benign, which necessitates the estimation of impact for all single variants. Clinical associations and experimental data are too limited for characterizing all variants, since more than 98% of the variants in human exomes have frequency of less than 1% (Karczewski et al. 2020; Van Hout et al. 2020) and over 40% of the ClinVar entries are catalogued as variants of unknown significance (Henrie et al. 2018). Protein function effect prediction methods have shown strong correlations with established associations and may be cautiously used to start bridging the gap in genome interpretation. With the advent of less costly sequencing technologies, clinicians can read patient’s genomes and search for precise therapies tailored to the genetic etiology of the disease. The insights provided by variant impact prediction methods may assist clinicians in selecting beneficial treatments.

## Data Availability

Not applicable.

## References

[CR100] Abecasis GR, Altshuler D, Auton A, Brooks LD, Durbin RM, Gibbs RA, Hurles ME, McVean GA, 1000 Genomes Project Consortium (2010). A map of human genome variation from population-scale sequencing. Nature.

[CR1] Abugessaisa I, Ramilowski JA, Lizio M, Severin J, Hasegawa A, Harshbarger J, Kondo A, Noguchi S, Yip CW, Ooi JLC, Tagami M, Hori F, Agrawal S, Hon CC, Cardon M, Ikeda S, Ono H, Bono H, Kato M, Hashimoto K, Bonetti A, Kato M, Kobayashi N, Shin J, de Hoon M, Hayashizaki Y, Carninci P, Kawaji H, Kasukawa T (2021). FANTOM enters 20th year: expansion of transcriptomic atlases and functional annotation of non-coding RNAs. Nucleic Acids Res.

[CR2] Adikesavan AK, Katsonis P, Marciano DC, Lua R, Herman C, Lichtarge O (2011). Separation of recombination and SOS response in Escherichia coli RecA suggests LexA interaction sites. PLoS Genet.

[CR3] Adzhubei IA, Schmidt S, Peshkin L, Ramensky VE, Gerasimova A, Bork P, Kondrashov AS, Sunyaev SR (2010). A method and server for predicting damaging missense mutations. Nat Methods.

[CR4] Ahmad S, Gromiha M, Fawareh H, Sarai A (2004). ASAView: database and tool for solvent accessibility representation in proteins. BMC Bioinform.

[CR5] Ainscough BJ, Griffith M, Coffman AC, Wagner AH, Kunisaki J, Choudhary MN, McMichael JF, Fulton RS, Wilson RK, Griffith OL, Mardis ER (2016). DoCM: a database of curated mutations in cancer. Nat Methods.

[CR6] Alirezaie N, Kernohan KD, Hartley T, Majewski J, Hocking TD (2018). ClinPred: prediction tool to identify disease-relevant nonsynonymous single-nucleotide variants. Am J Hum Genet.

[CR7] Denny JC, Rutter JL, Goldstein DB, Philippakis A, Smoller JW, Jenkins G, Dishman E, All of Us Research Program I (2019). The "All of Us" Research Program. N Engl J Med.

[CR8] Almannai M, Wang J, Dai H, El-Hattab AW, Faqeih EA, Saleh MA, Al Asmari A, Alwadei AH, Aljadhai YI, AlHashem A, Tabarki B, Lines MA, Grange DK, Benini R, Alsaman AS, Mahmoud A, Katsonis P, Lichtarge O, Wong LC (2018). FARS2 deficiency; new cases, review of clinical, biochemical, and molecular spectra, and variants interpretation based on structural, functional, and evolutionary significance. Mol Genet Metab.

[CR9] Ameur A, Dahlberg J, Olason P, Vezzi F, Karlsson R, Martin M, Viklund J, Kahari AK, Lundin P, Che H, Thutkawkorapin J, Eisfeldt J, Lampa S, Dahlberg M, Hagberg J, Jareborg N, Liljedahl U, Jonasson I, Johansson A, Feuk L, Lundeberg J, Syvanen AC, Lundin S, Nilsson D, Nystedt B, Magnusson PK, Gyllensten U (2017). SweGen: a whole-genome data resource of genetic variability in a cross-section of the Swedish population. Eur J Hum Genet.

[CR10] Andreoletti G, Pal LR, Moult J, Brenner SE (2019) Reports from the fifth edition of CAGI: The critical assessment of genome interpretation. Hum Mutat 40: 1197–1201. 10.1002/humu.23876 10.1002/humu.23876 PMC7329230 31334884

[CR11] Armon A, Graur D, Ben-Tal N (2001). ConSurf: an algorithmic tool for the identification of functional regions in proteins by surface mapping of phylogenetic information. J Mol Biol.

[CR12] Bacchelli E, Cainazzo MM, Cameli C, Guerzoni S, Martinelli A, Zoli M, Maestrini E, Pini LA (2016). A genome-wide analysis in cluster headache points to neprilysin and PACAP receptor gene variants. J Headache Pain.

[CR13] Backman JD, Li AH, Marcketta A, Sun D, Mbatchou J, Kessler MD, Benner C, Liu D, Locke AE, Balasubramanian S, Yadav A, Banerjee N, Gillies CE, Damask A, Liu S, Bai X, Hawes A, Maxwell E, Gurski L, Watanabe K, Kosmicki JA, Rajagopal V, Mighty J, Regeneron Genetics C, DiscovEhr JM, Mitnaul L, Stahl E, Coppola G, Jorgenson E, Habegger L, Salerno WJ, Shuldiner AR, Lotta LA, Overton JD, Cantor MN, Reid JG, Yancopoulos G, Kang HM, Marchini J, Baras A, Abecasis GR, Ferreira MAR (2021). Exome sequencing and analysis of 454,787 UK Biobank participants. Nature.

[CR14] Baek M, DiMaio F, Anishchenko I, Dauparas J, Ovchinnikov S, Lee GR, Wang J, Cong Q, Kinch LN, Schaeffer RD, Millan C, Park H, Adams C, Glassman CR, DeGiovanni A, Pereira JH, Rodrigues AV, van Dijk AA, Ebrecht AC, Opperman DJ, Sagmeister T, Buhlheller C, Pavkov-Keller T, Rathinaswamy MK, Dalwadi U, Yip CK, Burke JE, Garcia KC, Grishin NV, Adams PD, Read RJ, Baker D (2021). Accurate prediction of protein structures and interactions using a three-track neural network. Science.

[CR15] Bailey MH, Tokheim C, Porta-Pardo E, Sengupta S, Bertrand D, Weerasinghe A, Colaprico A, Wendl MC, Kim J, Reardon B, Ng PK, Jeong KJ, Cao S, Wang Z, Gao J, Gao Q, Wang F, Liu EM, Mularoni L, Rubio-Perez C, Nagarajan N, Cortes-Ciriano I, Zhou DC, Liang WW, Hess JM, Yellapantula VD, Tamborero D, Gonzalez-Perez A, Suphavilai C, Ko JY, Khurana E, Park PJ, Van Allen EM, Liang H, Group MCW, Cancer Genome Atlas Research N, Lawrence MS, Godzik A, Lopez-Bigas N, Stuart J, Wheeler D, Getz G, Chen K, Lazar AJ, Mills GB, Karchin R, Ding L (2018) Comprehensive characterization of cancer driver genes and mutations. Cell 173: 371–385 e18. 10.1016/j.cell.2018.02.060

[CR16] Bartha I, Rausell A, McLaren PJ, Mohammadi P, Tardaguila M, Chaturvedi N, Fellay J, Telenti A (2015). The characteristics of heterozygous protein truncating variants in the human genome. PLoS Comput Biol.

[CR17] Baugh EH, Simmons-Edler R, Muller CL, Alford RF, Volfovsky N, Lash AE, Bonneau R (2016). Robust classification of protein variation using structural modelling and large-scale data integration. Nucleic Acids Res.

[CR18] Beecham GW, Bis JC, Martin ER, Choi SH, DeStefano AL, van Duijn CM, Fornage M, Gabriel SB, Koboldt DC, Larson DE, Naj AC, Psaty BM, Salerno W, Bush WS, Foroud TM, Wijsman E, Farrer LA, Goate A, Haines JL, Pericak-Vance MA, Boerwinkle E, Mayeux R, Seshadri S, Schellenberg G (2017). The Alzheimer's disease sequencing project: study design and sample selection. Neurol Genet.

[CR19] Belonogova NM, Zorkoltseva IV, Tsepilov YA, Axenovich TI (2021). Gene-based association analysis identifies 190 genes affecting neuroticism. Sci Rep.

[CR20] Bendl J, Stourac J, Salanda O, Pavelka A, Wieben ED, Zendulka J, Brezovsky J, Damborsky J (2014). PredictSNP: robust and accurate consensus classifier for prediction of disease-related mutations. PLoS Comput Biol.

[CR21] Bendl J, Musil M, Stourac J, Zendulka J, Damborsky J, Brezovsky J (2016). PredictSNP2: a unified platform for accurately evaluating snp effects by exploiting the different characteristics of variants in distinct genomic regions. PLoS Comput Biol.

[CR22] Bergstrom A, McCarthy SA, Hui R, Almarri MA, Ayub Q, Danecek P, Chen Y, Felkel S, Hallast P, Kamm J, Blanche H, Deleuze JF, Cann H, Mallick S, Reich D, Sandhu MS, Skoglund P, Scally A, Xue Y, Durbin R, Tyler-Smith C (2020). Insights into human genetic variation and population history from 929 diverse genomes. Science.

[CR23] Berliner N, Teyra J, Colak R, Garcia Lopez S, Kim PM (2014). Combining structural modeling with ensemble machine learning to accurately predict protein fold stability and binding affinity effects upon mutation. PLoS One.

[CR24] Bhasuran B, Natarajan J (2018). Automatic extraction of gene-disease associations from literature using joint ensemble learning. PLoS One.

[CR25] Bocchini CE, Nahmod K, Katsonis P, Kim S, Kasembeli MM, Freeman A, Lichtarge O, Makedonas G, Tweardy DJ (2016). Protein stabilization improves STAT3 function in autosomal dominant hyper-IgE syndrome. Blood.

[CR26] Borecki IB, Province MA (2008). Genetic and genomic discovery using family studies. Circulation.

[CR27] Bromberg Y, Rost B (2007). SNAP: predict effect of non-synonymous polymorphisms on function. Nucleic Acids Res.

[CR28] Buja A, Volfovsky N, Krieger AM, Lord C, Lash AE, Wigler M, Iossifov I (2018). Damaging de novo mutations diminish motor skills in children on the autism spectrum. Proc Natl Acad Sci U S A.

[CR29] Bush WS, Moore JH (2012) Chapter 11: Genome-wide association studies. PLoS Comput Biol 8: e1002822. 10.1371/journal.pcbi.1002822 10.1371/journal.pcbi.1002822 PMC3531285 23300413

[CR30] Buske OJ, Manickaraj A, Mital S, Ray PN, Brudno M (2013). Identification of deleterious synonymous variants in human genomes. Bioinformatics.

[CR31] Bycroft C, Freeman C, Petkova D, Band G, Elliott LT, Sharp K, Motyer A, Vukcevic D, Delaneau O, O'Connell J, Cortes A, Welsh S, Young A, Effingham M, McVean G, Leslie S, Allen N, Donnelly P, Marchini J (2018). The UK Biobank resource with deep phenotyping and genomic data. Nature.

[CR32] Cai B, Li B, Kiga N, Thusberg J, Bergquist T, Chen YC, Niknafs N, Carter H, Tokheim C, Beleva-Guthrie V, Douville C, Bhattacharya R, Yeo HTG, Fan J, Sengupta S, Kim D, Cline M, Turner T, Diekhans M, Zaucha J, Pal LR, Cao C, Yu CH, Yin Y, Carraro M, Giollo M, Ferrari C, Leonardi E, Tosatto SCE, Bobe J, Ball M, Hoskins RA, Repo S, Church G, Brenner SE, Moult J, Gough J, Stanke M, Karchin R, Mooney SD (2017). Matching phenotypes to whole genomes: Lessons learned from four iterations of the personal genome project community challenges. Hum Mutat.

[CR33] Calabrese R, Capriotti E, Fariselli P, Martelli PL, Casadio R (2009). Functional annotations improve the predictive score of human disease-related mutations in proteins. Hum Mutat.

[CR34] Cancer Genome Atlas Research Network (2011). Integrated genomic analyses of ovarian carcinoma. Nature.

[CR35] Cancer Genome Atlas Research Network (2017). Comprehensive and Integrative Genomic Characterization of Hepatocellular Carcinoma. Cell.

[CR36] Capriotti E, Fariselli P, Casadio R (2005). I-Mutant2.0: predicting stability changes upon mutation from the protein sequence or structure. Nucleic Acids Res.

[CR37] Capriotti E, Calabrese R, Casadio R (2006). Predicting the insurgence of human genetic diseases associated to single point protein mutations with support vector machines and evolutionary information. Bioinformatics.

[CR38] Capriotti E, Fariselli P, Rossi I, Casadio R (2008). A three-state prediction of single point mutations on protein stability changes. BMC Bioinform.

[CR39] Capriotti E, Altman RB, Bromberg Y (2013). Collective judgment predicts disease-associated single nucleotide variants. BMC Genom.

[CR40] Cardoso JG, Andersen MR, Herrgard MJ, Sonnenschein N (2015). Analysis of genetic variation and potential applications in genome-scale metabolic modeling. Front Bioeng Biotechnol.

[CR41] Carraro M, Minervini G, Giollo M, Bromberg Y, Capriotti E, Casadio R, Dunbrack R, Elefanti L, Fariselli P, Ferrari C, Gough J, Katsonis P, Leonardi E, Lichtarge O, Menin C, Martelli PL, Niroula A, Pal LR, Repo S, Scaini MC, Vihinen M, Wei Q, Xu Q, Yang Y, Yin Y, Zaucha J, Zhao H, Zhou Y, Brenner SE, Moult J, Tosatto SCE (2017). Performance of in silico tools for the evaluation of p16INK4a (CDKN2A) variants in CAGI. Hum Mutat.

[CR42] Carraro M, Monzon AM, Chiricosta L, Reggiani F, Aspromonte MC, Bellini M, Pagel K, Jiang Y, Radivojac P, Kundu K, Pal LR, Yin Y, Limongelli I, Andreoletti G, Moult J, Wilson SJ, Katsonis P, Lichtarge O, Chen J, Wang Y, Hu Z, Brenner SE, Ferrari C, Murgia A, Tosatto SCE, Leonardi E (2019). Assessment of patient clinical descriptions and pathogenic variants from gene panel sequences in the CAGI-5 intellectual disability challenge. Hum Mutat.

[CR43] Carter H, Chen S, Isik L, Tyekucheva S, Velculescu VE, Kinzler KW, Vogelstein B, Karchin R (2009). Cancer-specific high-throughput annotation of somatic mutations: computational prediction of driver missense mutations. Cancer Res.

[CR44] Carter H, Douville C, Stenson PD, Cooper DN, Karchin R (2013). Identifying Mendelian disease genes with the variant effect scoring tool. BMC Genom.

[CR45] Castellana S, Mazza T (2013). Congruency in the prediction of pathogenic missense mutations: state-of-the-art web-based tools. Brief Bioinform.

[CR46] Cea-Rama I, Coscolin C, Katsonis P, Bargiela R, Golyshin PN, Lichtarge O, Ferrer M, Sanz-Aparicio J (2021). Structure and evolutionary trace-assisted screening of a residue swapping the substrate ambiguity and chiral specificity in an esterase. Comput Struct Biotechnol J.

[CR47] Chakravorty S, Hegde M (2018). Inferring the effect of genomic variation in the new era of genomics. Hum Mutat.

[CR48] Chan PA, Duraisamy S, Miller PJ, Newell JA, McBride C, Bond JP, Raevaara T, Ollila S, Nystrom M, Grimm AJ, Christodoulou J, Oetting WS, Greenblatt MS (2007). Interpreting missense variants: comparing computational methods in human disease genes CDKN2A, MLH1, MSH2, MECP2, and tyrosinase (TYR). Hum Mutat.

[CR49] Chandonia JM, Adhikari A, Carraro M, Chhibber A, Cutting GR, Fu Y, Gasparini A, Jones DT, Kramer A, Kundu K, Lam HYK, Leonardi E, Moult J, Pal LR, Searls DB, Shah S, Sunyaev S, Tosatto SCE, Yin Y, Buckley BA (2017). Lessons from the CAGI-4 Hopkins clinical panel challenge. Hum Mutat.

[CR50] Chasman D, Adams RM (2001). Predicting the functional consequences of non-synonymous single nucleotide polymorphisms: structure-based assessment of amino acid variation. J Mol Biol.

[CR51] Chaudhuri TK, Paul S (2006). Protein-misfolding diseases and chaperone-based therapeutic approaches. FEBS J.

[CR52] Chen L, Jin P, Qin ZS (2016). DIVAN: accurate identification of non-coding disease-specific risk variants using multi-omics profiles. Genome Biol.

[CR53] Chen S, Fragoza R, Klei L, Liu Y, Wang J, Roeder K, Devlin B, Yu H (2018). An interactome perturbation framework prioritizes damaging missense mutations for developmental disorders. Nat Genet.

[CR54] Chen H, Li J, Wang Y, Ng PK, Tsang YH, Shaw KR, Mills GB, Liang H (2020). Comprehensive assessment of computational algorithms in predicting cancer driver mutations. Genome Biol.

[CR55] Cheng J, Randall A, Baldi P (2006). Prediction of protein stability changes for single-site mutations using support vector machines. Proteins.

[CR56] Chennen K, Weber T, Lornage X, Kress A, Bohm J, Thompson J, Laporte J, Poch O (2020). MISTIC: A prediction tool to reveal disease-relevant deleterious missense variants. PLoS One.

[CR57] Choi Y, Sims GE, Murphy S, Miller JR, Chan AP (2012). Predicting the functional effect of amino acid substitutions and indels. PLoS One.

[CR58] Chun S, Fay JC (2009). Identification of deleterious mutations within three human genomes. Genome Res.

[CR59] Chun YS, Passot G, Yamashita S, Nusrat M, Katsonis P, Loree JM, Conrad C, Tzeng CD, Xiao L, Aloia TA, Eng C, Kopetz SE, Lichtarge O, Vauthey JN (2019). Deleterious effect of RAS and evolutionary high-risk TP53 double mutation in colorectal liver metastases. Ann Surg.

[CR60] Clark WT, Kasak L, Bakolitsa C, Hu Z, Andreoletti G, Babbi G, Bromberg Y, Casadio R, Dunbrack R, Folkman L, Ford CT, Jones D, Katsonis P, Kundu K, Lichtarge O, Martelli PL, Mooney SD, Nodzak C, Pal LR, Radivojac P, Savojardo C, Shi X, Zhou Y, Uppal A, Xu Q, Yin Y, Pejaver V, Wang M, Wei L, Moult J, Yu GK, Brenner SE, LeBowitz JH (2019). Assessment of predicted enzymatic activity of alpha-N-acetylglucosaminidase variants of unknown significance for CAGI 2016. Hum Mutat.

[CR61] Clarke L, Fairley S, Zheng-Bradley X, Streeter I, Perry E, Lowy E, Tasse AM, Flicek P (2017). The international genome sample resource (IGSR): A worldwide collection of genome variation incorporating the 1000 genomes project data. Nucleic Acids Res.

[CR62] Clarke CN, Katsonis P, Hsu TK, Koire AM, Silva-Figueroa A, Christakis I, Williams MD, Kutahyalioglu M, Kwatampora L, Xi Y, Lee JE, Koptez ES, Busaidy NL, Perrier ND, Lichtarge O (2019). Comprehensive genomic characterization of parathyroid cancer identifies novel candidate driver mutations and core pathways. J Endocr Soc.

[CR63] Cline MS, Karchin R (2011). Using bioinformatics to predict the functional impact of SNVs. Bioinformatics.

[CR64] Cline MS, Liao RG, Parsons MT, Paten B, Alquaddoomi F, Antoniou A, Baxter S, Brody L, Cook-Deegan R, Coffin A, Couch FJ, Craft B, Currie R, Dlott CC, Dolman L, den Dunnen JT, Dyke SOM, Domchek SM, Easton D, Fischmann Z, Foulkes WD, Garber J, Goldgar D, Goldman MJ, Goodhand P, Harrison S, Haussler D, Kato K, Knoppers B, Markello C, Nussbaum R, Offit K, Plon SE, Rashbass J, Rehm HL, Robson M, Rubinstein WS, Stoppa-Lyonnet D, Tavtigian S, Thorogood A, Zhang C, Zimmermann M, Authors BC, Burn J, Chanock S, Ratsch G, Spurdle AB (2018). BRCA challenge: BRCA exchange as a global resource for variants in BRCA1 and BRCA2. PLoS Genet.

[CR65] Cline MS, Babbi G, Bonache S, Cao Y, Casadio R, de la Cruz X, Diez O, Gutierrez-Enriquez S, Katsonis P, Lai C, Lichtarge O, Martelli PL, Mishne G, Moles-Fernandez A, Montalban G, Mooney SD, O'Conner R, Ootes L, Ozkan S, Padilla N, Pagel KA, Pejaver V, Radivojac P, Riera C, Savojardo C, Shen Y, Sun Y, Topper S, Parsons MT, Spurdle AB, Goldgar DE, Consortium E (2019) Assessment of blind predictions of the clinical significance of BRCA1 and BRCA2 variants. Hum Mutat 40: 1546–1556. 10.1002/humu.23861 10.1002/humu.23861 PMC6744348 31294896

[CR66] Cooper GM, Shendure J (2011). Needles in stacks of needles: finding disease-causal variants in a wealth of genomic data. Nat Rev Genet.

[CR67] Cooper DN, Krawczak M, Polychronakos C, Tyler-Smith C, Kehrer-Sawatzki H (2013). Where genotype is not predictive of phenotype: towards an understanding of the molecular basis of reduced penetrance in human inherited disease. Hum Genet.

[CR68] Cvijovic I, Good BH, Desai MM (2018). The effect of strong purifying selection on genetic diversity. Genetics.

[CR69] Daneshjou R, Wang Y, Bromberg Y, Bovo S, Martelli PL, Babbi G, Lena PD, Casadio R, Edwards M, Gifford D, Jones DT, Sundaram L, Bhat RR, Li X, Pal LR, Kundu K, Yin Y, Moult J, Jiang Y, Pejaver V, Pagel KA, Li B, Mooney SD, Radivojac P, Shah S, Carraro M, Gasparini A, Leonardi E, Giollo M, Ferrari C, Tosatto SCE, Bachar E, Azaria JR, Ofran Y, Unger R, Niroula A, Vihinen M, Chang B, Wang MH, Franke A, Petersen BS, Pirooznia M, Zandi P, McCombie R, Potash JB, Altman RB, Klein TE, Hoskins RA, Repo S, Brenner SE, Morgan AA (2017). Working toward precision medicine: predicting phenotypes from exomes in the critical assessment of genome interpretation (CAGI) challenges. Hum Mutat.

[CR70] Dash R, Mitra S, Munni YA, Choi HJ, Ali MC, Barua L, Jang TJ, Moon IS (2021). Computational insights into the deleterious impacts of missense variants on n-acetyl-d-glucosamine kinase structure and function. Int J Mol Sci.

[CR71] Davis CA, Hitz BC, Sloan CA, Chan ET, Davidson JM, Gabdank I, Hilton JA, Jain K, Baymuradov UK, Narayanan AK, Onate KC, Graham K, Miyasato SR, Dreszer TR, Strattan JS, Jolanki O, Tanaka FY, Cherry JM (2018). The encyclopedia of DNA elements (ENCODE): data portal update. Nucleic Acids Res.

[CR72] Davoli T, Xu AW, Mengwasser KE, Sack LM, Yoon JC, Park PJ, Elledge SJ (2013). Cumulative haploinsufficiency and triplosensitivity drive aneuploidy patterns and shape the cancer genome. Cell.

[CR73] Davydov EV, Goode DL, Sirota M, Cooper GM, Sidow A, Batzoglou S (2010). Identifying a high fraction of the human genome to be under selective constraint using GERP++. PLoS Comput Biol.

[CR74] Dehouck Y, Kwasigroch JM, Gilis D, Rooman M (2011). PoPMuSiC 2.1: a web server for the estimation of protein stability changes upon mutation and sequence optimality. BMC Bioinform.

[CR75] Denny JC, Ritchie MD, Basford MA, Pulley JM, Bastarache L, Brown-Gentry K, Wang D, Masys DR, Roden DM, Crawford DC (2010). PheWAS: demonstrating the feasibility of a phenome-wide scan to discover gene-disease associations. Bioinformatics.

[CR76] Desmet FO, Hamroun D, Lalande M, Collod-Beroud G, Claustres M, Beroud C (2009). Human splicing finder: an online bioinformatics tool to predict splicing signals. Nucleic Acids Res.

[CR77] Dietlein F, Weghorn D, Taylor-Weiner A, Richters A, Reardon B, Liu D, Lander ES, Van Allen EM, Sunyaev SR (2020). Identification of cancer driver genes based on nucleotide context. Nat Genet.

[CR78] DiGiammarino EL, Lee AS, Cadwell C, Zhang W, Bothner B, Ribeiro RC, Zambetti G, Kriwacki RW (2002). A novel mechanism of tumorigenesis involving pH-dependent destabilization of a mutant p53 tetramer. Nat Struct Biol.

[CR79] DNA sequencing costs: Data (2020) DNA Sequencing Costs: Data

[CR80] Dong C, Wei P, Jian X, Gibbs R, Boerwinkle E, Wang K, Liu X (2015). Comparison and integration of deleteriousness prediction methods for nonsynonymous SNVs in whole exome sequencing studies. Hum Mol Genet.

[CR81] Dousse A, Junier T, Zdobnov EM (2016). CEGA–a catalog of conserved elements from genomic alignments. Nucleic Acids Res.

[CR82] Douville C, Masica DL, Stenson PD, Cooper DN, Gygax DM, Kim R, Ryan M, Karchin R (2016). Assessing the pathogenicity of insertion and deletion variants with the variant effect scoring tool (VEST-Indel). Hum Mutat.

[CR83] Dunker AK, Brown CJ, Lawson JD, Iakoucheva LM, Obradovic Z (2002). Intrinsic disorder and protein function. Biochemistry.

[CR84] Encode Project Consortium (2012). An integrated encyclopedia of DNA elements in the human genome. Nature.

[CR85] Fariselli P, Martelli PL, Savojardo C, Casadio R (2015). INPS: predicting the impact of non-synonymous variations on protein stability from sequence. Bioinformatics.

[CR86] Fischbach GD, Lord C (2010). The Simons Simplex Collection: a resource for identification of autism genetic risk factors. Neuron.

[CR87] Flanagan SE, Patch AM, Ellard S (2010). Using SIFT and PolyPhen to predict loss-of-function and gain-of-function mutations. Genet Test Mol Biomarkers.

[CR88] Fokkema IF, den Dunnen JT, Taschner PE (2005). LOVD: easy creation of a locus-specific sequence variation database using an "LSDB-in-a-box" approach. Hum Mutat.

[CR89] Folkman L, Yang Y, Li Z, Stantic B, Sattar A, Mort M, Cooper DN, Liu Y, Zhou Y (2015). DDIG-in: detecting disease-causing genetic variations due to frameshifting indels and nonsense mutations employing sequence and structural properties at nucleotide and protein levels. Bioinformatics.

[CR90] French JD, Edwards SL (2020). The role of noncoding variants in heritable disease. Trends Genet.

[CR91] Fu W, Akey JM (2013). Selection and adaptation in the human genome. Annu Rev Genom Hum Genet.

[CR92] Fu Y, Liu Z, Lou S, Bedford J, Mu XJ, Yip KY, Khurana E, Gerstein M (2014). FunSeq2: a framework for prioritizing noncoding regulatory variants in cancer. Genome Biol.

[CR93] Gallagher MD, Chen-Plotkin AS (2018). The post-GWAS Era: from association to function. Am J Hum Genet.

[CR94] Gallion J, Koire A, Katsonis P, Schoenegge AM, Bouvier M, Lichtarge O (2017). Predicting phenotype from genotype: Improving accuracy through more robust experimental and computational modeling. Hum Mutat.

[CR95] Ganesan K, Kulandaisamy A, Binny Priya S, Gromiha MM (2019). HuVarBase: A human variant database with comprehensive information at gene and protein levels. PLoS One.

[CR96] Garber M, Guttman M, Clamp M, Zody MC, Friedman N, Xie X (2009). Identifying novel constrained elements by exploiting biased substitution patterns. Bioinformatics.

[CR97] Gelfman S, Wang Q, McSweeney KM, Ren Z, La Carpia F, Halvorsen M, Schoch K, Ratzon F, Heinzen EL, Boland MJ, Petrovski S, Goldstein DB (2017). Annotating pathogenic non-coding variants in genic regions. Nat Commun.

[CR98] Genome of the Netherlands Consortium (2014). Whole-genome sequence variation, population structure and demographic history of the Dutch population. Nat Genet.

[CR99] GenomeAsia 100K Consortium (2019). The GenomeAsia 100K Project enables genetic discoveries across Asia. Nature.

[CR101] Ghosh R, Oak N, Plon SE (2017). Evaluation of in silico algorithms for use with ACMG/AMP clinical variant interpretation guidelines. Genome Biol.

[CR102] Gibson SB, Downie JM, Tsetsou S, Feusier JE, Figueroa KP, Bromberg MB, Jorde LB, Pulst SM (2017). The evolving genetic risk for sporadic ALS. Neurology.

[CR103] Giollo M, Jones DT, Carraro M, Leonardi E, Ferrari C, Tosatto SCE (2017). Crohn disease risk prediction-Best practices and pitfalls with exome data. Hum Mutat.

[CR104] Glaser F, Pupko T, Paz I, Bell RE, Bechor-Shental D, Martz E, Ben-Tal N (2003). ConSurf: identification of functional regions in proteins by surface-mapping of phylogenetic information. Bioinformatics.

[CR105] Gomez-Cabrero D, Abugessaisa I, Maier D, Teschendorff A, Merkenschlager M, Gisel A, Ballestar E, Bongcam-Rudloff E, Conesa A, Tegner J (2014). Data integration in the era of omics: current and future challenges. BMC Syst Biol.

[CR106] Gonzalez-Perez A, Lopez-Bigas N (2011). Improving the assessment of the outcome of nonsynonymous SNVs with a consensus deleteriousness score, Condel. Am J Hum Genet.

[CR107] Gonzalez-Perez A, Lopez-Bigas N (2012). Functional impact bias reveals cancer drivers. Nucleic Acids Res.

[CR108] Gout AM, Martin NC, Brown AF, Ravine D (2007). PKDB: polycystic kidney disease mutation database–a gene variant database for autosomal dominant polycystic kidney disease. Hum Mutat.

[CR109] Grantham R (1974). Amino acid difference formula to help explain protein evolution. Science.

[CR110] Gray VE, Hause RJ, Fowler DM (2017). Analysis of large-scale mutagenesis data to assess the impact of single amino acid substitutions. Genetics.

[CR111] Gray VE, Hause RJ, Luebeck J, Shendure J, Fowler DM (2018). Quantitative missense variant effect prediction using large-scale mutagenesis data. Cell Syst.

[CR112] Greenman C, Stephens P, Smith R, Dalgliesh GL, Hunter C, Bignell G, Davies H, Teague J, Butler A, Stevens C, Edkins S, O'Meara S, Vastrik I, Schmidt EE, Avis T, Barthorpe S, Bhamra G, Buck G, Choudhury B, Clements J, Cole J, Dicks E, Forbes S, Gray K, Halliday K, Harrison R, Hills K, Hinton J, Jenkinson A, Jones D, Menzies A, Mironenko T, Perry J, Raine K, Richardson D, Shepherd R, Small A, Tofts C, Varian J, Webb T, West S, Widaa S, Yates A, Cahill DP, Louis DN, Goldstraw P, Nicholson AG, Brasseur F, Looijenga L, Weber BL, Chiew YE, DeFazio A, Greaves MF, Green AR, Campbell P, Birney E, Easton DF, Chenevix-Trench G, Tan MH, Khoo SK, Teh BT, Yuen ST, Leung SY, Wooster R, Futreal PA, Stratton MR (2007). Patterns of somatic mutation in human cancer genomes. Nature.

[CR113] Grimm DG, Azencott CA, Aicheler F, Gieraths U, MacArthur DG, Samocha KE, Cooper DN, Stenson PD, Daly MJ, Smoller JW, Duncan LE, Borgwardt KM (2015). The evaluation of tools used to predict the impact of missense variants is hindered by two types of circularity. Hum Mutat.

[CR114] Grover S, Del Greco MF, Stein CM, Ziegler A (2017). Mendelian randomization. Methods Mol Biol.

[CR115] Grunseich C, Sarkar N, Lu J, Owen M, Schindler A, Calabresi PA, Sumner CJ, Roda RH, Chaudhry V, Lloyd TE, Crawford TO, Subramony SH, Oh SJ, Richardson P, Tanji K, Kwan JY, Fischbeck KH, Mankodi A (2021). Improving the efficacy of exome sequencing at a quaternary care referral centre: novel mutations, clinical presentations and diagnostic challenges in rare neurogenetic diseases. J Neurol Neurosurg Psychiatry.

[CR116] Guerois R, Nielsen JE, Serrano L (2002). Predicting changes in the stability of proteins and protein complexes: a study of more than 1000 mutations. J Mol Biol.

[CR117] Gulko B, Hubisz MJ, Gronau I, Siepel A (2015). A method for calculating probabilities of fitness consequences for point mutations across the human genome. Nat Genet.

[CR118] Gunning AC, Fryer V, Fasham J, Crosby AH, Ellard S, Baple EL, Wright CF (2020). Assessing performance of pathogenicity predictors using clinically relevant variant datasets. J Med Genet.

[CR119] Gutierrez-Sacristan A, De Niz C, Kothari C, Kong SW, Mandl KD, Avillach P (2021). GenoPheno: cataloging large-scale phenotypic and next-generation sequencing data within human datasets. Brief Bioinform.

[CR120] Hassan MS, Shaalan AA, Dessouky MI, Abdelnaiem AE, ElHefnawi M (2019). A review study: Computational techniques for expecting the impact of non-synonymous single nucleotide variants in human diseases. Gene.

[CR121] Hecht M, Bromberg Y, Rost B (2015). Better prediction of functional effects for sequence variants. BMC Genom.

[CR122] Hendlich M, Rippmann F, Barnickel G (1997). LIGSITE: automatic and efficient detection of potential small molecule-binding sites in proteins. J Mol Graph Model.

[CR123] Henikoff S, Henikoff JG (1992). Amino acid substitution matrices from protein blocks. Proc Natl Acad Sci U S A.

[CR124] Henikoff JG, Henikoff S (1996). Using substitution probabilities to improve position-specific scoring matrices. Comput Appl Biosci.

[CR125] Henrie A, Hemphill SE, Ruiz-Schultz N, Cushman B, DiStefano MT, Azzariti D, Harrison SM, Rehm HL, Eilbeck K (2018). ClinVar Miner: Demonstrating utility of a Web-based tool for viewing and filtering ClinVar data. Hum Mutat.

[CR126] Hicks S, Wheeler DA, Plon SE, Kimmel M (2011). Prediction of missense mutation functionality depends on both the algorithm and sequence alignment employed. Hum Mutat.

[CR127] Hopf TA, Ingraham JB, Poelwijk FJ, Scharfe CP, Springer M, Sander C, Marks DS (2017). Mutation effects predicted from sequence co-variation. Nat Biotechnol.

[CR128] Hoskins RA, Repo S, Barsky D, Andreoletti G, Moult J, Brenner SE (2017). Reports from CAGI: The critical assessment of genome interpretation. Hum Mutat.

[CR129] Hsu TK, Asmussen JK, Koire AM, Choi BK, Gadhikar MA, Huh E, Lin CH, Konecki DM, Kim YW, Pickering C, Kimmel M, Donehower LA, Frederick MJ, Myers JN, Katsonis P, Lichtarge O (2022). A general calculus of fitness landscapes finds genes under selection in cancers. Genome Res.

[CR130] Hu J, Ng PC (2012). Predicting the effects of frameshifting indels. Genome Biol.

[CR131] Hu H, Huff CD, Moore B, Flygare S, Reese MG, Yandell M (2013). VAAST 2.0: improved variant classification and disease-gene identification using a conservation-controlled amino acid substitution matrix. Genet Epidemiol.

[CR132] Hu H, Coon H, Li M, Yandell M, Huff CD (2016). VARPRISM: incorporating variant prioritization in tests of de novo mutation association. Genome Med.

[CR133] Hu Z, Yu C, Furutsuki M, Andreoletti G, Ly M, Hoskins R, Adhikari AN, Brenner SE (2019). VIPdb, a genetic variant impact predictor database. Hum Mutat.

[CR134] Huang H, Chanda P, Alonso A, Bader JS, Arking DE (2011). Gene-based tests of association. PLoS Genet.

[CR135] Huang S, Chaudhary K, Garmire LX (2017). More Is better: recent progress in multi-omics data integration methods. Front Genet.

[CR136] Huang YF, Gulko B, Siepel A (2017). Fast, scalable prediction of deleterious noncoding variants from functional and population genomic data. Nat Genet.

[CR137] Ioannidis NM, Rothstein JH, Pejaver V, Middha S, McDonnell SK, Baheti S, Musolf A, Li Q, Holzinger E, Karyadi D, Cannon-Albright LA, Teerlink CC, Stanford JL, Isaacs WB, Xu J, Cooney KA, Lange EM, Schleutker J, Carpten JD, Powell IJ, Cussenot O, Cancel-Tassin G, Giles GG, MacInnis RJ, Maier C, Hsieh CL, Wiklund F, Catalona WJ, Foulkes WD, Mandal D, Eeles RA, Kote-Jarai Z, Bustamante CD, Schaid DJ, Hastie T, Ostrander EA, Bailey-Wilson JE, Radivojac P, Thibodeau SN, Whittemore AS, Sieh W (2016). REVEL: an ensemble method for predicting the pathogenicity of rare missense variants. Am J Hum Genet.

[CR138] Ionita-Laza I, McCallum K, Xu B, Buxbaum JD (2016). A spectral approach integrating functional genomic annotations for coding and noncoding variants. Nat Genet.

[CR139] Iossifov I, Ronemus M, Levy D, Wang Z, Hakker I, Rosenbaum J, Yamrom B, Lee YH, Narzisi G, Leotta A, Kendall J, Grabowska E, Ma B, Marks S, Rodgers L, Stepansky A, Troge J, Andrews P, Bekritsky M, Pradhan K, Ghiban E, Kramer M, Parla J, Demeter R, Fulton LL, Fulton RS, Magrini VJ, Ye K, Darnell JC, Darnell RB, Mardis ER, Wilson RK, Schatz MC, McCombie WR, Wigler M (2012). De novo gene disruptions in children on the autistic spectrum. Neuron.

[CR140] Isvoran A, Louet M, Vladoiu DL, Craciun D, Loriot MA, Villoutreix BO, Miteva MA (2017). Pharmacogenomics of the cytochrome P450 2C family: impacts of amino acid variations on drug metabolism. Drug Discov Today.

[CR141] Jagadeesh KA, Wenger AM, Berger MJ, Guturu H, Stenson PD, Cooper DN, Bernstein JA, Bejerano G (2016). M-CAP eliminates a majority of variants of uncertain significance in clinical exomes at high sensitivity. Nat Genet.

[CR142] Jaganathan K, Kyriazopoulou Panagiotopoulou S, McRae JF, Darbandi SF, Knowles D, Li YI, Kosmicki JA, Arbelaez J, Cui W, Schwartz GB, Chow ED, Kanterakis E, Gao H, Kia A, Batzoglou S, Sanders SJ, Farh KK (2019). Predicting splicing from primary sequence with deep learning. Cell.

[CR143] Jain A, Bhoyar RC, Pandhare K, Mishra A, Sharma D, Imran M, Senthivel V, Divakar MK, Rophina M, Jolly B, Batra A, Sharma S, Siwach S, Jadhao AG, Palande NV, Jha GN, Ashrafi N, Mishra PK, A KV, Jain S, Dash D, Kumar NS, Vanlallawma A, Sarma RJ, Chhakchhuak L, Kalyanaraman S, Mahadevan R, Kandasamy S, B MP, Rajagopal RE, J ER, P ND, Bajaj A, Gupta V, Mathew S, Goswami S, Mangla M, Prakash S, Joshi K, S S, Gajjar D, Soraisham R, Yadav R, Devi YS, Gupta A, Mukerji M, Ramalingam S, B KB, Scaria V, Sivasubbu S (2021) IndiGenomes: a comprehensive resource of genetic variants from over 1000 Indian genomes. Nucleic Acids Res 49: D1225–D1232. 10.1093/nar/gkaa923 10.1093/nar/gkaa923 PMC7778947 33095885

[CR144] Jeon S, Bhak Y, Choi Y, Jeon Y, Kim S, Jang J, Jang J, Blazyte A, Kim C, Kim Y, Shim J, Kim N, Kim YJ, Park SG, Kim J, Cho YS, Park Y, Kim HM, Kim BC, Park NH, Shin ES, Kim BC, Bolser D, Manica A, Edwards JS, Church G, Lee S, Bhak J (2020) Korean Genome Project: 1094 Korean personal genomes with clinical information. Sci Adv 6: eaaz7835. 10.1126/sciadv.aaz7835 10.1126/sciadv.aaz7835 PMC7385432 32766443

[CR145] Jin SC, Homsy J, Zaidi S, Lu Q, Morton S, DePalma SR, Zeng X, Qi H, Chang W, Sierant MC, Hung WC, Haider S, Zhang J, Knight J, Bjornson RD, Castaldi C, Tikhonoa IR, Bilguvar K, Mane SM, Sanders SJ, Mital S, Russell MW, Gaynor JW, Deanfield J, Giardini A, Porter GA, Srivastava D, Lo CW, Shen Y, Watkins WS, Yandell M, Yost HJ, Tristani-Firouzi M, Newburger JW, Roberts AE, Kim R, Zhao H, Kaltman JR, Goldmuntz E, Chung WK, Seidman JG, Gelb BD, Seidman CE, Lifton RP, Brueckner M (2017). Contribution of rare inherited and de novo variants in 2,871 congenital heart disease probands. Nat Genet.

[CR146] John SE, Antony D, Eaaswarkhanth M, Hebbar P, Channanath AM, Thomas D, Devarajan S, Tuomilehto J, Al-Mulla F, Alsmadi O, Thanaraj TA (2018). Assessment of coding region variants in Kuwaiti population: implications for medical genetics and population genomics. Sci Rep.

[CR147] Jones DT, Buchan DW, Cozzetto D, Pontil M (2012). PSICOV: precise structural contact prediction using sparse inverse covariance estimation on large multiple sequence alignments. Bioinformatics.

[CR148] Jumper J, Evans R, Pritzel A, Green T, Figurnov M, Ronneberger O, Tunyasuvunakool K, Bates R, Zidek A, Potapenko A, Bridgland A, Meyer C, Kohl SAA, Ballard AJ, Cowie A, Romera-Paredes B, Nikolov S, Jain R, Adler J, Back T, Petersen S, Reiman D, Clancy E, Zielinski M, Steinegger M, Pacholska M, Berghammer T, Bodenstein S, Silver D, Vinyals O, Senior AW, Kavukcuoglu K, Kohli P, Hassabis D (2021). Highly accurate protein structure prediction with AlphaFold. Nature.

[CR149] Jung KS, Hong KW, Jo HY, Choi J, Ban HJ, Cho SB, Chung M (2020). KRGDB: the large-scale variant database of 1722 Koreans based on whole genome sequencing. Database (oxford).

[CR150] Kabsch W, Sander C (1983). Dictionary of protein secondary structure: pattern recognition of hydrogen-bonded and geometrical features. Biopolymers.

[CR151] Kaminker JS, Zhang Y, Watanabe C, Zhang Z (2007). CanPredict: a computational tool for predicting cancer-associated missense mutations. Nucleic Acids Res.

[CR152] Kanagal-Shamanna R, Montalban-Bravo G, Katsonis P, Sasaki K, Class CA, Jabbour E, Sallman D, Hunter AM, Benton C, Chien KS, Luthra R, Bueso-Ramos CE, Kadia T, Andreeff M, Komrokji RS, Al Ali NH, Short N, Daver N, Routbort MJ, Khoury JD, Patel K, Ganan-Gomez I, Wei Y, Borthakur G, Ravandi F, Do KA, Soltysiak KA, Lichtarge O, Medeiros LJ, Kantarjian H, Garcia-Manero G (2021). Evolutionary action score identifies a subset of TP53 mutated myelodysplastic syndrome with favorable prognosis. Blood Cancer J.

[CR153] Karczewski KJ, Weisburd B, Thomas B, Solomonson M, Ruderfer DM, Kavanagh D, Hamamsy T, Lek M, Samocha KE, Cummings BB, Birnbaum D, The Exome Aggregation C, Daly MJ, MacArthur DG (2017). The ExAC browser: displaying reference data information from over 60 000 exomes. Nucleic Acids Res.

[CR154] Karczewski KJ, Francioli LC, Tiao G, Cummings BB, Alfoldi J, Wang Q, Collins RL, Laricchia KM, Ganna A, Birnbaum DP, Gauthier LD, Brand H, Solomonson M, Watts NA, Rhodes D, Singer-Berk M, England EM, Seaby EG, Kosmicki JA, Walters RK, Tashman K, Farjoun Y, Banks E, Poterba T, Wang A, Seed C, Whiffin N, Chong JX, Samocha KE, Pierce-Hoffman E, Zappala Z, O'Donnell-Luria AH, Minikel EV, Weisburd B, Lek M, Ware JS, Vittal C, Armean IM, Bergelson L, Cibulskis K, Connolly KM, Covarrubias M, Donnelly S, Ferriera S, Gabriel S, Gentry J, Gupta N, Jeandet T, Kaplan D, Llanwarne C, Munshi R, Novod S, Petrillo N, Roazen D, Ruano-Rubio V, Saltzman A, Schleicher M, Soto J, Tibbetts K, Tolonen C, Wade G, Talkowski ME, Genome Aggregation Database C, Neale BM, Daly MJ, MacArthur DG (2020). The mutational constraint spectrum quantified from variation in 141,456 humans. Nature.

[CR155] Kasak L, Bakolitsa C, Hu Z, Yu C, Rine J, Dimster-Denk DF, Pandey G, De Baets G, Bromberg Y, Cao C, Capriotti E, Casadio R, Van Durme J, Giollo M, Karchin R, Katsonis P, Leonardi E, Lichtarge O, Martelli PL, Masica D, Mooney SD, Olatubosun A, Radivojac P, Rousseau F, Pal LR, Savojardo C, Schymkowitz J, Thusberg J, Tosatto SCE, Vihinen M, Valiaho J, Repo S, Moult J, Brenner SE, Friedberg I (2019). Assessing computational predictions of the phenotypic effect of cystathionine-beta-synthase variants. Hum Mutat.

[CR156] Kasak L, Hunter JM, Udani R, Bakolitsa C, Hu Z, Adhikari AN, Babbi G, Casadio R, Gough J, Guerrero RF, Jiang Y, Joseph T, Katsonis P, Kotte S, Kundu K, Lichtarge O, Martelli PL, Mooney SD, Moult J, Pal LR, Poitras J, Radivojac P, Rao A, Sivadasan N, Sunderam U, Saipradeep VG, Yin Y, Zaucha J, Brenner SE, Meyn MS (2019). CAGI SickKids challenges: Assessment of phenotype and variant predictions derived from clinical and genomic data of children with undiagnosed diseases. Hum Mutat.

[CR157] Kato S, Han SY, Liu W, Otsuka K, Shibata H, Kanamaru R, Ishioka C (2003). Understanding the function-structure and function-mutation relationships of p53 tumor suppressor protein by high-resolution missense mutation analysis. Proc Natl Acad Sci U S A.

[CR158] Katsonis P, Lichtarge O (2014). A formal perturbation equation between genotype and phenotype determines the Evolutionary Action of protein-coding variations on fitness. Genome Res.

[CR159] Katsonis P, Lichtarge O (2017). Objective assessment of the evolutionary action equation for the fitness effect of missense mutations across CAGI-blinded contests. Hum Mutat.

[CR160] Katsonis P, Lichtarge O (2019). CAGI5: Objective performance assessments of predictions based on the Evolutionary Action equation. Hum Mutat.

[CR161] Katsonis P, Koire A, Wilson SJ, Hsu TK, Lua RC, Wilkins AD, Lichtarge O (2014). Single nucleotide variations: biological impact and theoretical interpretation. Protein Sci.

[CR162] Kawabata T, Ota M, Nishikawa K (1999). The protein mutant database. Nucleic Acids Res.

[CR163] Kenna KP, van Doormaal PT, Dekker AM, Ticozzi N, Kenna BJ, Diekstra FP, van Rheenen W, van Eijk KR, Jones AR, Keagle P, Shatunov A, Sproviero W, Smith BN, van Es MA, Topp SD, Kenna A, Miller JW, Fallini C, Tiloca C, McLaughlin RL, Vance C, Troakes C, Colombrita C, Mora G, Calvo A, Verde F, Al-Sarraj S, King A, Calini D, de Belleroche J, Baas F, van der Kooi AJ, de Visser M, Ten Asbroek AL, Sapp PC, McKenna-Yasek D, Polak M, Asress S, Munoz-Blanco JL, Strom TM, Meitinger T, Morrison KE, Consortium S, Lauria G, Williams KL, Leigh PN, Nicholson GA, Blair IP, Leblond CS, Dion PA, Rouleau GA, Pall H, Shaw PJ, Turner MR, Talbot K, Taroni F, Boylan KB, Van Blitterswijk M, Rademakers R, Esteban-Perez J, Garcia-Redondo A, Van Damme P, Robberecht W, Chio A, Gellera C, Drepper C, Sendtner M, Ratti A, Glass JD, Mora JS, Basak NA, Hardiman O, Ludolph AC, Andersen PM, Weishaupt JH, Brown RH, Jr., Al-Chalabi A, Silani V, Shaw CE, van den Berg LH, Veldink JH, Landers JE (2016) NEK1 variants confer susceptibility to amyotrophic lateral sclerosis. Nat Genet 48: 1037-42. 10.1038/ng.3626 10.1038/ng.3626 PMC5560030 27455347

[CR164] Kim J, Weber JA, Jho S, Jang J, Jun J, Cho YS, Kim HM, Kim H, Kim Y, Chung O, Kim CG, Lee H, Kim BC, Han K, Koh I, Chae KS, Lee S, Edwards JS, Bhak J (2018). KoVariome: Korean national standard reference variome database of whole genomes with comprehensive SNV, indel, CNV, and SV analyses. Sci Rep.

[CR165] Kim YW, Al-Ramahi I, Koire A, Wilson SJ, Konecki DM, Mota S, Soleimani S, Botas J, Lichtarge O (2021). Harnessing the paradoxical phenotypes of APOE varepsilon2 and APOE varepsilon4 to identify genetic modifiers in Alzheimer's disease. Alzheimers Dement.

[CR166] Kimura M (1979). The neutral theory of molecular evolution. Sci Am.

[CR167] Kircher M, Witten DM, Jain P, O'Roak BJ, Cooper GM, Shendure J (2014). A general framework for estimating the relative pathogenicity of human genetic variants. Nat Genet.

[CR168] Koire A, Katsonis P, Kim YW, Buchovecky C, Wilson SJ, Lichtarge O (2021). A method to delineate de novo missense variants across pathways prioritizes genes linked to autism. Sci Transl Med.

[CR169] Kroos M, Pomponio RJ, van Vliet L, Palmer RE, Phipps M, Van der Helm R, Halley D, Reuser A, Consortium GAAD (2008) Update of the Pompe disease mutation database with 107 sequence variants and a format for severity rating. Hum Mutat 29: E13-26. 10.1002/humu.20745 10.1002/humu.20745 18425781

[CR170] Kumar P, Henikoff S, Ng PC (2009). Predicting the effects of coding non-synonymous variants on protein function using the SIFT algorithm. Nat Protoc.

[CR171] Kumar M, Gaharwar U, Paul S, Poojary M, Pandhare K, Scaria V, Bk B (2020). WilsonGen a comprehensive clinically annotated genomic variant resource for Wilson's Disease. Sci Rep.

[CR172] Landrum MJ, Lee JM, Benson M, Brown GR, Chao C, Chitipiralla S, Gu B, Hart J, Hoffman D, Jang W, Karapetyan K, Katz K, Liu C, Maddipatla Z, Malheiro A, McDaniel K, Ovetsky M, Riley G, Zhou G, Holmes JB, Kattman BL, Maglott DR (2018). ClinVar: improving access to variant interpretations and supporting evidence. Nucleic Acids Res.

[CR173] Lawrence MS, Stojanov P, Polak P, Kryukov GV, Cibulskis K, Sivachenko A, Carter SL, Stewart C, Mermel CH, Roberts SA, Kiezun A, Hammerman PS, McKenna A, Drier Y, Zou L, Ramos AH, Pugh TJ, Stransky N, Helman E, Kim J, Sougnez C, Ambrogio L, Nickerson E, Shefler E, Cortes ML, Auclair D, Saksena G, Voet D, Noble M, DiCara D, Lin P, Lichtenstein L, Heiman DI, Fennell T, Imielinski M, Hernandez B, Hodis E, Baca S, Dulak AM, Lohr J, Landau DA, Wu CJ, Melendez-Zajgla J, Hidalgo-Miranda A, Koren A, McCarroll SA, Mora J, Crompton B, Onofrio R, Parkin M, Winckler W, Ardlie K, Gabriel SB, Roberts CWM, Biegel JA, Stegmaier K, Bass AJ, Garraway LA, Meyerson M, Golub TR, Gordenin DA, Sunyaev S, Lander ES, Getz G (2013). Mutational heterogeneity in cancer and the search for new cancer-associated genes. Nature.

[CR174] Lee B, Richards FM (1971). The interpretation of protein structures: estimation of static accessibility. J Mol Biol.

[CR175] Lee S, Abecasis GR, Boehnke M, Lin X (2014). Rare-variant association analysis: study designs and statistical tests. Am J Hum Genet.

[CR176] Lees-Miller JP, Cobban A, Katsonis P, Bacolla A, Tsutakawa SE, Hammel M, Meek K, Anderson DW, Lichtarge O, Tainer JA, Lees-Miller SP (2021). Uncovering DNA-PKcs ancient phylogeny, unique sequence motifs and insights for human disease. Prog Biophys Mol Biol.

[CR177] Lek M, Karczewski KJ, Minikel EV, Samocha KE, Banks E, Fennell T, O'Donnell-Luria AH, Ware JS, Hill AJ, Cummings BB, Tukiainen T, Birnbaum DP, Kosmicki JA, Duncan LE, Estrada K, Zhao F, Zou J, Pierce-Hoffman E, Berghout J, Cooper DN, Deflaux N, DePristo M, Do R, Flannick J, Fromer M, Gauthier L, Goldstein J, Gupta N, Howrigan D, Kiezun A, Kurki MI, Moonshine AL, Natarajan P, Orozco L, Peloso GM, Poplin R, Rivas MA, Ruano-Rubio V, Rose SA, Ruderfer DM, Shakir K, Stenson PD, Stevens C, Thomas BP, Tiao G, Tusie-Luna MT, Weisburd B, Won HH, Yu D, Altshuler DM, Ardissino D, Boehnke M, Danesh J, Donnelly S, Elosua R, Florez JC, Gabriel SB, Getz G, Glatt SJ, Hultman CM, Kathiresan S, Laakso M, McCarroll S, McCarthy MI, McGovern D, McPherson R, Neale BM, Palotie A, Purcell SM, Saleheen D, Scharf JM, Sklar P, Sullivan PF, Tuomilehto J, Tsuang MT, Watkins HC, Wilson JG, Daly MJ, MacArthur DG, Exome Aggregation C (2016). Analysis of protein-coding genetic variation in 60,706 humans. Nature.

[CR178] Leman R, Gaildrat P, Le Gac G, Ka C, Fichou Y, Audrezet MP, Caux-Moncoutier V, Caputo SM, Boutry-Kryza N, Leone M, Mazoyer S, Bonnet-Dorion F, Sevenet N, Guillaud-Bataille M, Rouleau E, Bressac-de Paillerets B, Wappenschmidt B, Rossing M, Muller D, Bourdon V, Revillon F, Parsons MT, Rousselin A, Davy G, Castelain G, Castera L, Sokolowska J, Coulet F, Delnatte C, Ferec C, Spurdle AB, Martins A, Krieger S, Houdayer C (2018). Novel diagnostic tool for prediction of variant spliceogenicity derived from a set of 395 combined in silico/in vitro studies: an international collaborative effort. Nucleic Acids Res.

[CR179] Leong IU, Stuckey A, Lai D, Skinner JR, Love DR (2015). Assessment of the predictive accuracy of five in silico prediction tools, alone or in combination, and two metaservers to classify long QT syndrome gene mutations. BMC Med Genet.

[CR180] Li B, Krishnan VG, Mort ME, Xin F, Kamati KK, Cooper DN, Mooney SD, Radivojac P (2009). Automated inference of molecular mechanisms of disease from amino acid substitutions. Bioinformatics.

[CR181] Li J, Duncan DT, Zhang B (2010). CanProVar: a human cancer proteome variation database. Hum Mutat.

[CR182] Li Z, Gonzalez CL, Wang B, Zhang Y, Mejia O, Katsonis P, Lichtarge O, Myers JN, El-Naggar AK, Caulin C (2016). Cdkn2a suppresses metastasis in squamous cell carcinomas induced by the gain-of-function mutant p53(R172H). J Pathol.

[CR183] Li J, Zhao T, Zhang Y, Zhang K, Shi L, Chen Y, Wang X, Sun Z (2018). Performance evaluation of pathogenicity-computation methods for missense variants. Nucleic Acids Res.

[CR370] Li S, van der Velde KJ, de Ridder D, van Dijk ADJ, Soudis D, Zwerwer LR, Deelen P, Hendriksen D, Charbon B, van Gijn ME, Abbott K, Sikkema-Raddatz B, van Diemen CC, Kerstjens-Frederikse WS, Sinke RJ, Swertz MA (2020). CAPICE: a computational method for Consequence-Agnostic Pathogenicity Interpretation of Clinical Exome variations. Genome Med.

[CR184] Lichtarge O, Bourne HR, Cohen FE (1996). An evolutionary trace method defines binding surfaces common to protein families. J Mol Biol.

[CR185] Lin M, Whitmire S, Chen J, Farrel A, Shi X, Guo JT (2017). Effects of short indels on protein structure and function in human genomes. Sci Rep.

[CR186] Liu JZ, McRae AF, Nyholt DR, Medland SE, Wray NR, Brown KM, Investigators A, Hayward NK, Montgomery GW, Visscher PM, Martin NG, Macgregor S (2010). A versatile gene-based test for genome-wide association studies. Am J Hum Genet.

[CR187] Liu L, Sanderford MD, Patel R, Chandrashekar P, Gibson G, Kumar S (2019). Biological relevance of computationally predicted pathogenicity of noncoding variants. Nat Commun.

[CR188] Livesey BJ, Marsh JA (2020). Using deep mutational scanning to benchmark variant effect predictors and identify disease mutations. Mol Syst Biol.

[CR189] Livingstone M, Folkman L, Yang Y, Zhang P, Mort M, Cooper DN, Liu Y, Stantic B, Zhou Y (2017). Investigating DNA-, RNA-, and protein-based features as a means to discriminate pathogenic synonymous variants. Hum Mutat.

[CR190] Loeb DD, Swanstrom R, Everitt L, Manchester M, Stamper SE, Hutchison CA (1989). Complete mutagenesis of the HIV-1 protease. Nature.

[CR191] Loots GG, Ovcharenko I (2004). rVISTA 2.0: evolutionary analysis of transcription factor binding sites. Nucleic Acids Res.

[CR192] Lord C, Elsabbagh M, Baird G, Veenstra-Vanderweele J (2018). Autism spectrum disorder. Lancet.

[CR193] Lord C, Brugha TS, Charman T, Cusack J, Dumas G, Frazier T, Jones EJH, Jones RM, Pickles A, State MW, Taylor JL, Veenstra-VanderWeele J (2020). Autism spectrum disorder. Nat Rev Dis Primers.

[CR194] Lu Q, Hu Y, Sun J, Cheng Y, Cheung KH, Zhao H (2015). A statistical framework to predict functional non-coding regions in the human genome through integrated analysis of annotation data. Sci Rep.

[CR195] MacArthur DG, Tyler-Smith C (2010). Loss-of-function variants in the genomes of healthy humans. Hum Mol Genet.

[CR196] MacArthur J, Bowler E, Cerezo M, Gil L, Hall P, Hastings E, Junkins H, McMahon A, Milano A, Morales J, Pendlington ZM, Welter D, Burdett T, Hindorff L, Flicek P, Cunningham F, Parkinson H (2017). The new NHGRI-EBI Catalog of published genome-wide association studies (GWAS Catalog). Nucleic Acids Res.

[CR197] Mahmood K, Jung CH, Philip G, Georgeson P, Chung J, Pope BJ, Park DJ (2017). Variant effect prediction tools assessed using independent, functional assay-based datasets: implications for discovery and diagnostics. Hum Genomics.

[CR198] Mailman MD, Feolo M, Jin Y, Kimura M, Tryka K, Bagoutdinov R, Hao L, Kiang A, Paschall J, Phan L, Popova N, Pretel S, Ziyabari L, Lee M, Shao Y, Wang ZY, Sirotkin K, Ward M, Kholodov M, Zbicz K, Beck J, Kimelman M, Shevelev S, Preuss D, Yaschenko E, Graeff A, Ostell J, Sherry ST (2007). The NCBI dbGaP database of genotypes and phenotypes. Nat Genet.

[CR199] Malaria Genomic Epidemiology Network (2019). Insights into malaria susceptibility using genome-wide data on 17,000 individuals from Africa. Asia and Oceania Nat Commun.

[CR200] Mallick S, Li H, Lipson M, Mathieson I, Gymrek M, Racimo F, Zhao M, Chennagiri N, Nordenfelt S, Tandon A, Skoglund P, Lazaridis I, Sankararaman S, Fu Q, Rohland N, Renaud G, Erlich Y, Willems T, Gallo C, Spence JP, Song YS, Poletti G, Balloux F, van Driem G, de Knijff P, Romero IG, Jha AR, Behar DM, Bravi CM, Capelli C, Hervig T, Moreno-Estrada A, Posukh OL, Balanovska E, Balanovsky O, Karachanak-Yankova S, Sahakyan H, Toncheva D, Yepiskoposyan L, Tyler-Smith C, Xue Y, Abdullah MS, Ruiz-Linares A, Beall CM, Di Rienzo A, Jeong C, Starikovskaya EB, Metspalu E, Parik J, Villems R, Henn BM, Hodoglugil U, Mahley R, Sajantila A, Stamatoyannopoulos G, Wee JT, Khusainova R, Khusnutdinova E, Litvinov S, Ayodo G, Comas D, Hammer MF, Kivisild T, Klitz W, Winkler CA, Labuda D, Bamshad M, Jorde LB, Tishkoff SA, Watkins WS, Metspalu M, Dryomov S, Sukernik R, Singh L, Thangaraj K, Paabo S, Kelso J, Patterson N, Reich D (2016). The Simons Genome Diversity Project: 300 genomes from 142 diverse populations. Nature.

[CR201] Marciano DC, Lua RC, Katsonis P, Amin SR, Herman C, Lichtarge O (2014). Negative feedback in genetic circuits confers evolutionary resilience and capacitance. Cell Rep.

[CR202] Marek K, Chowdhury S, Siderowf A, Lasch S, Coffey CS, Caspell-Garcia C, Simuni T, Jennings D, Tanner CM, Trojanowski JQ, Shaw LM, Seibyl J, Schuff N, Singleton A, Kieburtz K, Toga AW, Mollenhauer B, Galasko D, Chahine LM, Weintraub D, Foroud T, Tosun-Turgut D, Poston K, Arnedo V, Frasier M, Sherer T, Parkinson's Progression Markers I (2018). The Parkinson's progression markers initiative (PPMI) - establishing a PD biomarker cohort. Ann Clin Transl Neurol.

[CR203] Markiewicz W, Amikam S, Roguin N, Riss E (1975). Changing haemodynamics in patient with papillary muscle dysfunction. Br Heart J.

[CR204] Markiewicz P, Kleina LG, Cruz C, Ehret S, Miller JH (1994). Genetic studies of the lac repressor. XIV. Analysis of 4000 altered Escherichia coli lac repressors reveals essential and non-essential residues, as well as "spacers" which do not require a specific sequence. J Mol Biol.

[CR205] Mathe E, Olivier M, Kato S, Ishioka C, Hainaut P, Tavtigian SV (2006). Computational approaches for predicting the biological effect of p53 missense mutations: a comparison of three sequence analysis based methods. Nucleic Acids Res.

[CR206] Matimba A, Del-Favero J, Van Broeckhoven C, Masimirembwa C (2009). Novel variants of major drug-metabolising enzyme genes in diverse African populations and their predicted functional effects. Hum Genomics.

[CR207] McInnes G, Daneshjou R, Katsonis P, Lichtarge O, Srinivasan R, Rana S, Radivojac P, Mooney SD, Pagel KA, Stamboulian M, Jiang Y, Capriotti E, Wang Y, Bromberg Y, Bovo S, Savojardo C, Martelli PL, Casadio R, Pal LR, Moult J, Brenner SE, Altman R (2019). Predicting venous thromboembolism risk from exomes in the Critical Assessment of Genome Interpretation (CAGI) challenges. Hum Mutat.

[CR208] Michels M, Matte U, Fraga LR, Mancuso ACB, Ligabue-Braun R, Berneira EFR, Siebert M, Sanseverino MTV (2019). Determining the pathogenicity of CFTR missense variants: Multiple comparisons of in silico predictors and variant annotation databases. Genet Mol Biol.

[CR209] Mihalek I, Res I, Lichtarge O (2004). A family of evolution-entropy hybrid methods for ranking protein residues by importance. J Mol Biol.

[CR210] Miller DT, Adam MP, Aradhya S, Biesecker LG, Brothman AR, Carter NP, Church DM, Crolla JA, Eichler EE, Epstein CJ, Faucett WA, Feuk L, Friedman JM, Hamosh A, Jackson L, Kaminsky EB, Kok K, Krantz ID, Kuhn RM, Lee C, Ostell JM, Rosenberg C, Scherer SW, Spinner NB, Stavropoulos DJ, Tepperberg JH, Thorland EC, Vermeesch JR, Waggoner DJ, Watson MS, Martin CL, Ledbetter DH (2010). Consensus statement: chromosomal microarray is a first-tier clinical diagnostic test for individuals with developmental disabilities or congenital anomalies. Am J Hum Genet.

[CR211] Miller M, Wang Y, Bromberg Y (2019). What went wrong with variant effect predictor performance for the PCM1 challenge. Hum Mutat.

[CR212] Miosge LA, Field MA, Sontani Y, Cho V, Johnson S, Palkova A, Balakishnan B, Liang R, Zhang Y, Lyon S, Beutler B, Whittle B, Bertram EM, Enders A, Goodnow CC, Andrews TD (2015). Comparison of predicted and actual consequences of missense mutations. Proc Natl Acad Sci U S A.

[CR213] Monplaisir N, Merault G, Poyart C, Rhoda MD, Craescu C, Vidaud M, Galacteros F, Blouquit Y, Rosa J (1986). Hemoglobin S Antilles: a variant with lower solubility than hemoglobin S and producing sickle cell disease in heterozygotes. Proc Natl Acad Sci U S A.

[CR214] Montanucci L, Capriotti E, Frank Y, Ben-Tal N, Fariselli P (2019). DDGun: an untrained method for the prediction of protein stability changes upon single and multiple point variations. BMC Bioinformatics.

[CR215] Monzon AM, Carraro M, Chiricosta L, Reggiani F, Han J, Ozturk K, Wang Y, Miller M, Bromberg Y, Capriotti E, Savojardo C, Babbi G, Martelli PL, Casadio R, Katsonis P, Lichtarge O, Carter H, Kousi M, Katsanis N, Andreoletti G, Moult J, Brenner SE, Ferrari C, Leonardi E, Tosatto SCE (2019). Performance of computational methods for the evaluation of pericentriolar material 1 missense variants in CAGI-5. Hum Mutat.

[CR216] Morcos F, Pagnani A, Lunt B, Bertolino A, Marks DS, Sander C, Zecchina R, Onuchic JN, Hwa T, Weigt M (2011). Direct-coupling analysis of residue coevolution captures native contacts across many protein families. Proc Natl Acad Sci U S A.

[CR217] Mort M, Sterne-Weiler T, Li B, Ball EV, Cooper DN, Radivojac P, Sanford JR, Mooney SD (2014). MutPred Splice: machine learning-based prediction of exonic variants that disrupt splicing. Genome Biol.

[CR218] Mullaney JM, Mills RE, Pittard WS, Devine SE (2010). Small insertions and deletions (INDELs) in human genomes. Hum Mol Genet.

[CR219] Mullany LK, Wong KK, Marciano DC, Katsonis P, King-Crane ER, Ren YA, Lichtarge O, Richards JS (2015). Specific TP53 mutants overrepresented in ovarian cancer impact CNV, TP53 Activity, Responses to nutlin-3a, and cell survival. Neoplasia.

[CR220] Munro D, Singh M (2020). DeMaSk: a deep mutational scanning substitution matrix and its use for variant impact prediction. Bioinformatics.

[CR221] Nagasaki M, Yasuda J, Katsuoka F, Nariai N, Kojima K, Kawai Y, Yamaguchi-Kabata Y, Yokozawa J, Danjoh I, Saito S, Sato Y, Mimori T, Tsuda K, Saito R, Pan X, Nishikawa S, Ito S, Kuroki Y, Tanabe O, Fuse N, Kuriyama S, Kiyomoto H, Hozawa A, Minegishi N, Douglas Engel J, Kinoshita K, Kure S, Yaegashi N, To MJRPP, Yamamoto M (2015). Rare variant discovery by deep whole-genome sequencing of 1,070 Japanese individuals. Nat Commun.

[CR222] Nair A, Chung HC, Sun T, Tyagi S, Dobrolecki LE, Dominguez-Vidana R, Kurley SJ, Orellana M, Renwick A, Henke DM, Katsonis P, Schmitt E, Chan DW, Li H, Mao S, Petrovic I, Creighton CJ, Gutierrez C, Dubrulle J, Stossi F, Tyner JW, Lichtarge O, Lin CY, Zhang B, Scott KL, Hilsenbeck SG, Sun J, Yu X, Osborne CK, Schiff R, Christensen JG, Shields DJ, Rimawi MF, Ellis MJ, Shaw CA, Lewis MT, Westbrook TF (2018). Combinatorial inhibition of PTPN12-regulated receptors leads to a broadly effective therapeutic strategy in triple-negative breast cancer. Nat Med.

[CR223] Neskey DM, Osman AA, Ow TJ, Katsonis P, McDonald T, Hicks SC, Hsu TK, Pickering CR, Ward A, Patel A, Yordy JS, Skinner HD, Giri U, Sano D, Story MD, Beadle BM, El-Naggar AK, Kies MS, William WN, Caulin C, Frederick M, Kimmel M, Myers JN, Lichtarge O (2015). Evolutionary Action Score of TP53 Identifies High-Risk Mutations Associated with Decreased Survival and Increased Distant Metastases in Head and Neck Cancer. Cancer Res.

[CR224] Ng PC, Henikoff S (2001). Predicting deleterious amino acid substitutions. Genome Res.

[CR225] Ng PC, Henikoff S (2003). SIFT: Predicting amino acid changes that affect protein function. Nucleic Acids Res.

[CR226] NHLBI Exome Sequencing Project (2011) NHLBI Exome Sequencing Project

[CR227] Niroula A, Urolagin S, Vihinen M (2015). PON-P2: prediction method for fast and reliable identification of harmful variants. PLoS ONE.

[CR228] Olatubosun A, Valiaho J, Harkonen J, Thusberg J, Vihinen M (2012). PON-P: integrated predictor for pathogenicity of missense variants. Hum Mutat.

[CR229] Oluwole OG, Kuivaniemi H, Abrahams S, Haylett WL, Vorster AA, van Heerden CJ, Kenyon CP, Tabb DL, Fawale MB, Sunmonu TA, Ajose A, Olaogun MO, Rossouw AC, van Hillegondsberg LS, Carr J, Ross OA, Komolafe MA, Tromp G, Bardien S (2020). Targeted next-generation sequencing identifies novel variants in candidate genes for Parkinson's disease in Black South African and Nigerian patients. BMC Med Genet.

[CR230] Orengo CA, Todd AE, Thornton JM (1999). From protein structure to function. Curr Opin Struct Biol.

[CR231] Osman AA, Monroe MM, Ortega Alves MV, Patel AA, Katsonis P, Fitzgerald AL, Neskey DM, Frederick MJ, Woo SH, Caulin C, Hsu TK, McDonald TO, Kimmel M, Meyn RE, Lichtarge O, Myers JN (2015). Wee-1 kinase inhibition overcomes cisplatin resistance associated with high-risk TP53 mutations in head and neck cancer through mitotic arrest followed by senescence. Mol Cancer Ther.

[CR232] Osman AA, Neskey DM, Katsonis P, Patel AA, Ward AM, Hsu TK, Hicks SC, McDonald TO, Ow TJ, Alves MO, Pickering CR, Skinner HD, Zhao M, Sturgis EM, Kies MS, El-Naggar A, Perrone F, Licitra L, Bossi P, Kimmel M, Frederick MJ, Lichtarge O, Myers JN (2015). Evolutionary action score of TP53 coding variants is predictive of platinum response in head and neck cancer patients. Cancer Res.

[CR233] Otaify GA, Whyte MP, Gottesman GS, McAlister WH, Eric Gordon J, Hollander A, Andrews MV, El-Mofty SK, Chen WS, Veis DV, Stolina M, Woo AS, Katsonis P, Lichtarge O, Zhang F, Shinawi M (2018). Gnathodiaphyseal dysplasia: Severe atypical presentation with novel heterozygous mutation of the anoctamin gene (ANO5). Bone.

[CR234] Pachkov M, Balwierz PJ, Arnold P, Ozonov E, van Nimwegen E (2013). SwissRegulon, a database of genome-wide annotations of regulatory sites: recent updates. Nucleic Acids Res.

[CR235] Pagel KA, Pejaver V, Lin GN, Nam HJ, Mort M, Cooper DN, Sebat J, Iakoucheva LM, Mooney SD, Radivojac P (2017). When loss-of-function is loss of function: assessing mutational signatures and impact of loss-of-function genetic variants. Bioinformatics.

[CR236] Pagel KA, Antaki D, Lian A, Mort M, Cooper DN, Sebat J, Iakoucheva LM, Mooney SD, Radivojac P (2019). Pathogenicity and functional impact of non-frameshifting insertion/deletion variation in the human genome. PLoS Comput Biol.

[CR237] Pal LR, Kundu K, Yin Y, Moult J (2017). CAGI4 Crohn's exome challenge: Marker SNP versus exome variant models for assigning risk of Crohn disease. Hum Mutat.

[CR238] Pal LR, Kundu K, Yin Y, Moult J (2020). Matching whole genomes to rare genetic disorders: Identification of potential causative variants using phenotype-weighted knowledge in the CAGI SickKids5 clinical genomes challenge. Hum Mutat.

[CR239] Parthiban V, Gromiha MM, Schomburg D (2006). CUPSAT: prediction of protein stability upon point mutations. Nucleic Acids Res.

[CR240] Parvandeh S, Donehower LA, Katsonis P, Hsu TK, Asmussen JK, Lee K, Lichtarge O (2022). EPIMUTESTR: a nearest neighbor machine learning approach to predict cancer driver genes from the evolutionary action of coding variants. Nucleic Acids Res.

[CR241] Patwa Z, Wahl LM (2008). The fixation probability of beneficial mutations. J R Soc Interface.

[CR368] Pei J, Kinch LN, Otwinowski Z, Grishin NV (2020). Mutation severity spectrum of rare alleles in the human genome is predictive of disease type. PLoS Comput Biol.

[CR242] Pejaver V, Mooney SD, Radivojac P (2017). Missense variant pathogenicity predictors generalize well across a range of function-specific prediction challenges. Hum Mutat.

[CR243] Pejaver V, Urresti J, Lugo-Martinez J, Pagel KA, Lin GN, Nam HJ, Mort M, Cooper DN, Sebat J, Iakoucheva LM, Mooney SD, Radivojac P (2020). Inferring the molecular and phenotypic impact of amino acid variants with MutPred2. Nat Commun.

[CR244] Peltomaki P, Vasen H (2004). Mutations associated with HNPCC predisposition – Update of ICG-HNPCC/INSiGHT mutation database. Dis Markers.

[CR245] Pereira R, Oliveira J, Sousa M (2020). Bioinformatics and computational tools for next-generation sequencing analysis in clinical genetics. J Clin Med.

[CR246] Petersen RC, Aisen PS, Beckett LA, Donohue MC, Gamst AC, Harvey DJ, Jack CR, Jagust WJ, Shaw LM, Toga AW, Trojanowski JQ, Weiner MW (2010). Alzheimer's disease neuroimaging initiative (ADNI): clinical characterization. Neurology.

[CR247] Peterson SM, Pack TF, Wilkins AD, Urs NM, Urban DJ, Bass CE, Lichtarge O, Caron MG (2015). Elucidation of G-protein and beta-arrestin functional selectivity at the dopamine D2 receptor. Proc Natl Acad Sci U S A.

[CR248] Petitjean A, Mathe E, Kato S, Ishioka C, Tavtigian SV, Hainaut P, Olivier M (2007). Impact of mutant p53 functional properties on TP53 mutation patterns and tumor phenotype: lessons from recent developments in the IARC TP53 database. Hum Mutat.

[CR249] Petrovski S, Wang Q, Heinzen EL, Allen AS, Goldstein DB (2013). Genic intolerance to functional variation and the interpretation of personal genomes. PLoS Genet.

[CR250] Pey AL, Stricher F, Serrano L, Martinez A (2007). Predicted effects of missense mutations on native-state stability account for phenotypic outcome in phenylketonuria, a paradigm of misfolding diseases. Am J Hum Genet.

[CR251] Pires DE, Ascher DB, Blundell TL (2014). DUET: a server for predicting effects of mutations on protein stability using an integrated computational approach. Nucleic Acids Res.

[CR252] Plotkin JB, Kudla G (2011). Synonymous but not the same: the causes and consequences of codon bias. Nat Rev Genet.

[CR253] Pollard KS, Hubisz MJ, Rosenbloom KR, Siepel A (2010). Detection of nonneutral substitution rates on mammalian phylogenies. Genome Res.

[CR371] Ponzoni L, Peñaherrera DA, Oltvai ZN, Bahar I, Ponty Y (2020). Rhapsody: predicting the pathogenicity of human missense variants. Bioinformatics.

[CR254] Pritchard JK, Przeworski M (2001). Linkage disequilibrium in humans: models and data. Am J Hum Genet.

[CR255] Pupko T, Bell RE, Mayrose I, Glaser F, Ben-Tal N (2002). Rate4Site: an algorithmic tool for the identification of functional regions in proteins by surface mapping of evolutionary determinants within their homologues. Bioinformatics.

[CR256] Qi H, Zhang H, Zhao Y, Chen C, Long JJ, Chung WK, Guan Y, Shen Y (2021). MVP predicts the pathogenicity of missense variants by deep learning. Nat Commun.

[CR257] Quan L, Lv Q, Zhang Y (2016). STRUM: structure-based prediction of protein stability changes upon single-point mutation. Bioinformatics.

[CR258] Quang D, Chen Y, Xie X (2015). DANN: a deep learning approach for annotating the pathogenicity of genetic variants. Bioinformatics.

[CR369] Quinodoz M, Peter VG, Cisarova K, Royer-Bertrand B, Stenson PD, Cooper DN, Unger S, Superti-Furga A, Rivolta C (2022). Analysis of missense variants in the human genome reveals widespread gene-specific clustering and improves prediction of pathogenicity. Am J Hum Genet.

[CR259] Rababa'h A, Craft JW, Wijaya CS, Atrooz F, Fan Q, Singh S, Guillory AN, Katsonis P, Lichtarge O, McConnell BK (2013). Protein kinase A and phosphodiesterase-4D3 binding to coding polymorphisms of cardiac muscle anchoring protein (mAKAP). J Mol Biol.

[CR260] Raimondi D, Gazzo AM, Rooman M, Lenaerts T, Vranken WF (2016). Multilevel biological characterization of exomic variants at the protein level significantly improves the identification of their deleterious effects. Bioinformatics.

[CR261] Raimondi D, Tanyalcin I, Ferte J, Gazzo A, Orlando G, Lenaerts T, Rooman M, Vranken W (2017). DEOGEN2: prediction and interactive visualization of single amino acid variant deleteriousness in human proteins. Nucleic Acids Res.

[CR262] Ramensky V, Bork P, Sunyaev S (2002). Human non-synonymous SNPs: server and survey. Nucleic Acids Res.

[CR263] Rehm HL, Berg JS, Brooks LD, Bustamante CD, Evans JP, Landrum MJ, Ledbetter DH, Maglott DR, Martin CL, Nussbaum RL, Plon SE, Ramos EM, Sherry ST, Watson MS, ClinGen (2015). ClinGen–the clinical genome resource. N Engl J Med.

[CR264] Rennell D, Bouvier SE, Hardy LW, Poteete AR (1991). Systematic mutation of bacteriophage T4 lysozyme. J Mol Biol.

[CR265] Rentzsch P, Schubach M, Shendure J, Kircher M (2021). CADD-Splice-improving genome-wide variant effect prediction using deep learning-derived splice scores. Genome Med.

[CR266] Reva B, Antipin Y, Sander C (2011). Predicting the functional impact of protein mutations: application to cancer genomics. Nucleic Acids Res.

[CR267] Richards S, Aziz N, Bale S, Bick D, Das S, Gastier-Foster J, Grody WW, Hegde M, Lyon E, Spector E, Voelkerding K, Rehm HL, Committee ALQA (2015). Standards and guidelines for the interpretation of sequence variants: a joint consensus recommendation of the American College of medical genetics and genomics and the association for molecular pathology. Genet Med.

[CR268] Risch NJ (2000). Searching for genetic determinants in the new millennium. Nature.

[CR269] Ritchie GR, Dunham I, Zeggini E, Flicek P (2014). Functional annotation of noncoding sequence variants. Nat Methods.

[CR270] Rivera-Munoz EA, Milko LV, Harrison SM, Azzariti DR, Kurtz CL, Lee K, Mester JL, Weaver MA, Currey E, Craigen W, Eng C, Funke B, Hegde M, Hershberger RE, Mao R, Steiner RD, Vincent LM, Martin CL, Plon SE, Ramos E, Rehm HL, Watson M, Berg JS (2018). ClinGen variant curation expert panel experiences and standardized processes for disease and gene-level specification of the ACMG/AMP guidelines for sequence variant interpretation. Hum Mutat.

[CR271] Rodriguez GJ, Yao R, Lichtarge O, Wensel TG (2010). Evolution-guided discovery and recoding of allosteric pathway specificity determinants in psychoactive bioamine receptors. Proc Natl Acad Sci U S A.

[CR272] Rogers MF, Shihab HA, Mort M, Cooper DN, Gaunt TR, Campbell C (2018). FATHMM-XF: accurate prediction of pathogenic point mutations via extended features. Bioinformatics.

[CR273] Rosenthal LS, Drake D, Alcalay RN, Babcock D, Bowman FD, Chen-Plotkin A, Dawson TM, Dewey RB, German DC, Huang X, Landin B, McAuliffe M, Petyuk VA, Scherzer CR, Hillaire-Clarke CS, Sieber BA, Sutherland M, Tarn C, West A, Vaillancourt D, Zhang J, Gwinn K, Consortium P (2016). The NINDS Parkinson's disease biomarkers program. Mov Disord.

[CR274] Salinas VH, Ranganathan R (2018). Coevolution-based inference of amino acid interactions underlying protein function. Elife.

[CR275] Samocha KE, Kosmicki JA, Karczewski KJ, O’Donnell-Luria AH, Pierce-Hoffman E, MacArthur DG, Neale BM, M.J. D,  (2017). Regional missense constraint improves variant deleteriousness prediction. bioRxiv.

[CR276] Sasidharan Nair P, Vihinen M (2013). VariBench: a benchmark database for variations. Hum Mutat.

[CR277] Schaaf CP, Koster J, Katsonis P, Kratz L, Shchelochkov OA, Scaglia F, Kelley RI, Lichtarge O, Waterham HR, Shinawi M (2011). Desmosterolosis-phenotypic and molecular characterization of a third case and review of the literature. Am J Med Genet A.

[CR278] Schonegge AM, Gallion J, Picard LP, Wilkins AD, Le Gouill C, Audet M, Stallaert W, Lohse MJ, Kimmel M, Lichtarge O, Bouvier M (2017). Evolutionary action and structural basis of the allosteric switch controlling beta2AR functional selectivity. Nat Commun.

[CR279] Schwarz JM, Rodelsperger C, Schuelke M, Seelow D (2010). MutationTaster evaluates disease-causing potential of sequence alterations. Nat Methods.

[CR280] Schwarz JM, Cooper DN, Schuelke M, Seelow D (2014). MutationTaster2: mutation prediction for the deep-sequencing age. Nat Methods.

[CR281] Sevim Bayrak C, Zhang P, Tristani-Firouzi M, Gelb BD, Itan Y (2020). De novo variants in exomes of congenital heart disease patients identify risk genes and pathways. Genome Med.

[CR282] Sharma A, Biswas A, Liu H, Sen S, Paruchuri A, Katsonis P, Lichtarge O, Chand Dakal T, Maulik U, Gromiha MM, Bandyopadhyay S, Ludwig M, Holz FG, Loeffler KU, Herwig-Carl MC (2019). Mutational landscape of the BAP1 locus reveals an intrinsic control to regulate the miRNA network and the binding of protein complexes in uveal melanoma. Cancers (basel).

[CR283] Shen W, Li Y (2016). A novel algorithm for detecting multiple covariance and clustering of biological sequences. Sci Rep.

[CR284] Shen H, Li J, Zhang J, Xu C, Jiang Y, Wu Z, Zhao F, Liao L, Chen J, Lin Y, Tian Q, Papasian CJ, Deng HW (2013). Comprehensive characterization of human genome variation by high coverage whole-genome sequencing of forty four Caucasians. PLoS One.

[CR285] Shi Z, Moult J (2011). Structural and functional impact of cancer-related missense somatic mutations. J Mol Biol.

[CR286] Shi F, Yao Y, Bin Y, Zheng CH, Xia J (2019). Computational identification of deleterious synonymous variants in human genomes using a feature-based approach. BMC Med Genom.

[CR287] Shihab HA, Gough J, Cooper DN, Stenson PD, Barker GL, Edwards KJ, Day IN, Gaunt TR (2013). Predicting the functional, molecular, and phenotypic consequences of amino acid substitutions using hidden Markov models. Hum Mutat.

[CR288] Shihab HA, Rogers MF, Gough J, Mort M, Cooper DN, Day IN, Gaunt TR, Campbell C (2015). An integrative approach to predicting the functional effects of non-coding and coding sequence variation. Bioinformatics.

[CR289] Siekierska A, De Baets G, Reumers J, Gallardo R, Rudyak S, Broersen K, Couceiro J, Van Durme J, Schymkowitz J, Rousseau F (2012). alpha-Galactosidase aggregation is a determinant of pharmacological chaperone efficacy on Fabry disease mutants. J Biol Chem.

[CR290] Siepel A, Bejerano G, Pedersen JS, Hinrichs AS, Hou M, Rosenbloom K, Clawson H, Spieth J, Hillier LW, Richards S, Weinstock GM, Wilson RK, Gibbs RA, Kent WJ, Miller W, Haussler D (2005). Evolutionarily conserved elements in vertebrate, insect, worm, and yeast genomes. Genome Res.

[CR291] Somody JC, MacKinnon SS, Windemuth A (2017). Structural coverage of the proteome for pharmaceutical applications. Drug Discov Today.

[CR292] Spurdle AB, Couch FJ, Hogervorst FB, Radice P, Sinilnikova OM, Group IUGVW (2008). Prediction and assessment of splicing alterations: implications for clinical testing. Hum Mutat.

[CR293] Sruthi CK, Prakash M (2020). Deep2Full: Evaluating strategies for selecting the minimal mutational experiments for optimal computational predictions of deep mutational scan outcomes. PLoS ONE.

[CR294] Sruthi CK, Balaram H, Prakash MK (2020). Toward Developing Intuitive Rules for Protein Variant Effect Prediction Using Deep Mutational Scanning Data. ACS Omega.

[CR295] Stavropoulos DJ, Merico D, Jobling R, Bowdin S, Monfared N, Thiruvahindrapuram B, Nalpathamkalam T, Pellecchia G, Yuen RKC, Szego MJ, Hayeems RZ, Shaul RZ, Brudno M, Girdea M, Frey B, Alipanahi B, Ahmed S, Babul-Hirji R, Porras RB, Carter MT, Chad L, Chaudhry A, Chitayat D, Doust SJ, Cytrynbaum C, Dupuis L, Ejaz R, Fishman L, Guerin A, Hashemi B, Helal M, Hewson S, Inbar-Feigenberg M, Kannu P, Karp N, Kim R, Kronick J, Liston E, MacDonald H, Mercimek-Mahmutoglu S, Mendoza-Londono R, Nasr E, Nimmo G, Parkinson N, Quercia N, Raiman J, Roifman M, Schulze A, Shugar A, Shuman C, Sinajon P, Siriwardena K, Weksberg R, Yoon G, Carew C, Erickson R, Leach RA, Klein R, Ray PN, Meyn MS, Scherer SW, Cohn RD, Marshall CR (2016). Whole genome sequencing expands diagnostic utility and improves clinical management in pediatric medicine. NPJ Genom Med.

[CR296] Stefl S, Nishi H, Petukh M, Panchenko AR, Alexov E (2013). Molecular mechanisms of disease-causing missense mutations. J Mol Biol.

[CR297] Stenson PD, Mort M, Ball EV, Chapman M, Evans K, Azevedo L, Hayden M, Heywood S, Millar DS, Phillips AD, Cooper DN (2020). The human gene mutation database (HGMD((R))): optimizing its use in a clinical diagnostic or research setting. Hum Genet.

[CR298] Stone EA, Sidow A (2005). Physicochemical constraint violation by missense substitutions mediates impairment of protein function and disease severity. Genome Res.

[CR299] Stratton MR, Campbell PJ, Futreal PA (2009). The cancer genome. Nature.

[CR300] Subramanian I, Verma S, Kumar S, Jere A, Anamika K (2020). Multi-omics data integration, interpretation, and its application. Bioinform Biol Insights.

[CR301] Sudlow C, Gallacher J, Allen N, Beral V, Burton P, Danesh J, Downey P, Elliott P, Green J, Landray M, Liu B, Matthews P, Ong G, Pell J, Silman A, Young A, Sprosen T, Peakman T, Collins R (2015). UK biobank: an open access resource for identifying the causes of a wide range of complex diseases of middle and old age. PLoS Med.

[CR302] Sukhai MA, Craddock KJ, Thomas M, Hansen AR, Zhang T, Siu L, Bedard P, Stockley TL, Kamel-Reid S (2016). A classification system for clinical relevance of somatic variants identified in molecular profiling of cancer. Genet Med.

[CR303] Sun Z, Liu Q, Qu G, Feng Y, Reetz MT (2019). Utility of B-factors in protein science: interpreting rigidity, flexibility, and internal motion and engineering thermostability. Chem Rev.

[CR304] Sundaram L, Gao H, Padigepati SR, McRae JF, Li Y, Kosmicki JA, Fritzilas N, Hakenberg J, Dutta A, Shon J, Xu J, Batzoglou S, Li X, Farh KK (2018). Predicting the clinical impact of human mutation with deep neural networks. Nat Genet.

[CR305] Sunyaev SR, Eisenhaber F, Rodchenkov IV, Eisenhaber B, Tumanyan VG, Kuznetsov EN (1999). PSIC: profile extraction from sequence alignments with position-specific counts of independent observations. Protein Eng.

[CR306] Suryavanshi SV, Jadhav SM, Anderson KL, Katsonis P, Lichtarge O, McConnell BK (2018). Human muscle-specific A-kinase anchoring protein polymorphisms modulate the susceptibility to cardiovascular diseases by altering cAMP/PKA signaling. Am J Physiol Heart Circ Physiol.

[CR307] Suybeng V, Koeppel F, Harle A, Rouleau E (2020). Comparison of Pathogenicity Prediction Tools on Somatic Variants. J Mol Diagn.

[CR308] Takeda JI, Nanatsue K, Yamagishi R, Ito M, Haga N, Hirata H, Ogi T, Ohno K (2020). InMeRF: prediction of pathogenicity of missense variants by individual modeling for each amino acid substitution. NAR Genom Bioinform.

[CR309] Taliun D, Harris DN, Kessler MD, Carlson J, Szpiech ZA, Torres R, Taliun SAG, Corvelo A, Gogarten SM, Kang HM, Pitsillides AN, LeFaive J, Lee SB, Tian X, Browning BL, Das S, Emde AK, Clarke WE, Loesch DP, Shetty AC, Blackwell TW, Smith AV, Wong Q, Liu X, Conomos MP, Bobo DM, Aguet F, Albert C, Alonso A, Ardlie KG, Arking DE, Aslibekyan S, Auer PL, Barnard J, Barr RG, Barwick L, Becker LC, Beer RL, Benjamin EJ, Bielak LF, Blangero J, Boehnke M, Bowden DW, Brody JA, Burchard EG, Cade BE, Casella JF, Chalazan B, Chasman DI, Chen YI, Cho MH, Choi SH, Chung MK, Clish CB, Correa A, Curran JE, Custer B, Darbar D, Daya M, de Andrade M, DeMeo DL, Dutcher SK, Ellinor PT, Emery LS, Eng C, Fatkin D, Fingerlin T, Forer L, Fornage M, Franceschini N, Fuchsberger C, Fullerton SM, Germer S, Gladwin MT, Gottlieb DJ, Guo X, Hall ME, He J, Heard-Costa NL, Heckbert SR, Irvin MR, Johnsen JM, Johnson AD, Kaplan R, Kardia SLR, Kelly T, Kelly S, Kenny EE, Kiel DP, Klemmer R, Konkle BA, Kooperberg C, Kottgen A, Lange LA, Lasky-Su J, Levy D, Lin X, Lin KH, Liu C, Loos RJF (2021). Sequencing of 53,831 diverse genomes from the NHLBI TOPMed Program. Nature.

[CR310] Tamborero D, Gonzalez-Perez A, Lopez-Bigas N (2013). OncodriveCLUST: exploiting the positional clustering of somatic mutations to identify cancer genes. Bioinformatics.

[CR311] Tang H, Thomas PD (2016). Tools for predicting the functional impact of nonsynonymous genetic variation. Genetics.

[CR312] Tate JG, Bamford S, Jubb HC, Sondka Z, Beare DM, Bindal N, Boutselakis H, Cole CG, Creatore C, Dawson E, Fish P, Harsha B, Hathaway C, Jupe SC, Kok CY, Noble K, Ponting L, Ramshaw CC, Rye CE, Speedy HE, Stefancsik R, Thompson SL, Wang S, Ward S, Campbell PJ, Forbes SA (2019). COSMIC: the catalogue of somatic mutations in cancer. Nucleic Acids Res.

[CR313] Tavtigian SV, Deffenbaugh AM, Yin L, Judkins T, Scholl T, Samollow PB, de Silva D, Zharkikh A, Thomas A (2006). Comprehensive statistical study of 452 BRCA1 missense substitutions with classification of eight recurrent substitutions as neutral. J Med Genet.

[CR314] Tchernitchko D, Goossens M, Wajcman H (2004). In silico prediction of the deleterious effect of a mutation: proceed with caution in clinical genetics. Clin Chem.

[CR315] Teng S, Madej T, Panchenko A, Alexov E (2009). Modeling effects of human single nucleotide polymorphisms on protein-protein interactions. Biophys J.

[CR316] Teng S, Sobitan A, Rhoades R, Liu D, Tang Q (2021). Systemic effects of missense mutations on SARS-CoV-2 spike glycoprotein stability and receptor-binding affinity. Brief Bioinform.

[CR317] The 1000 Genomes Project Consortium (2015). A global reference for human genetic variation. Nature.

[CR318] The International HapMap Consortium (2003). The International HapMap Project. Nature.

[CR319] Thomas PD, Campbell MJ, Kejariwal A, Mi H, Karlak B, Daverman R, Diemer K, Muruganujan A, Narechania A (2003). PANTHER: a library of protein families and subfamilies indexed by function. Genome Res.

[CR320] Tian Y, Pesaran T, Chamberlin A, Fenwick RB, Li S, Gau CL, Chao EC, Lu HM, Black MH, Qian D (2019). REVEL and BayesDel outperform other in silico meta-predictors for clinical variant classification. Sci Rep.

[CR321] Uversky VN, Oldfield CJ, Dunker AK (2008). Intrinsically disordered proteins in human diseases: introducing the D2 concept. Annu Rev Biophys.

[CR322] van Dam S, Vosa U, van der Graaf A, Franke L, de Magalhaes JP (2018). Gene co-expression analysis for functional classification and gene-disease predictions. Brief Bioinform.

[CR323] Van Hout CV, Tachmazidou I, Backman JD, Hoffman JD, Liu D, Pandey AK, Gonzaga-Jauregui C, Khalid S, Ye B, Banerjee N, Li AH, O'Dushlaine C, Marcketta A, Staples J, Schurmann C, Hawes A, Maxwell E, Barnard L, Lopez A, Penn J, Habegger L, Blumenfeld AL, Bai X, O'Keeffe S, Yadav A, Praveen K, Jones M, Salerno WJ, Chung WK, Surakka I, Willer CJ, Hveem K, Leader JB, Carey DJ, Ledbetter DH, Geisinger-Regeneron Discov EHRC, Cardon L, Yancopoulos GD, Economides A, Coppola G, Shuldiner AR, Balasubramanian S, Cantor M, Regeneron Genetics C, Nelson MR, Whittaker J, Reid JG, Marchini J, Overton JD, Scott RA, Abecasis GR, Yerges-Armstrong L, Baras A (2020). Exome sequencing and characterization of 49,960 individuals in the UK Biobank. Nature.

[CR324] Vardarajan BN, Ghani M, Kahn A, Sheikh S, Sato C, Barral S, Lee JH, Cheng R, Reitz C, Lantigua R, Reyes-Dumeyer D, Medrano M, Jimenez-Velazquez IZ, Rogaeva E, St George-Hyslop P, Mayeux R (2015). Rare coding mutations identified by sequencing of Alzheimer disease genome-wide association studies loci. Ann Neurol.

[CR325] Vaser R, Adusumalli S, Leng SN, Sikic M, Ng PC (2016). SIFT missense predictions for genomes. Nat Protoc.

[CR326] Voskanian A, Katsonis P, Lichtarge O, Pejaver V, Radivojac P, Mooney SD, Capriotti E, Bromberg Y, Wang Y, Miller M, Martelli PL, Savojardo C, Babbi G, Casadio R, Cao Y, Sun Y, Shen Y, Garg A, Pal D, Yu Y, Huff CD, Tavtigian SV, Young E, Neuhausen SL, Ziv E, Pal LR, Andreoletti G, Brenner SE, Kann MG (2019). Assessing the performance of in silico methods for predicting the pathogenicity of variants in the gene CHEK2, among Hispanic females with breast cancer. Hum Mutat.

[CR327] Waalen J, Beutler E (2009). Genetic screening for low-penetrance variants in protein-coding genes. Annu Rev Genomics Hum Genet.

[CR328] Walters-Sen LC, Hashimoto S, Thrush DL, Reshmi S, Gastier-Foster JM, Astbury C, Pyatt RE (2015). Variability in pathogenicity prediction programs: impact on clinical diagnostics. Mol Genet Genomic Med.

[CR329] Wang Y, Bromberg Y (2019). Identifying mutation-driven changes in gene functionality that lead to venous thromboembolism. Hum Mutat.

[CR330] Wang Z, Moult J (2001). SNPs, protein structure, and disease. Hum Mutat.

[CR331] Wang T, Zhan X, Bu CH, Lyon S, Pratt D, Hildebrand S, Choi JH, Zhang Z, Zeng M, Wang KW, Turer E, Chen Z, Zhang D, Yue T, Wang Y, Shi H, Wang J, Sun L, SoRelle J, McAlpine W, Hutchins N, Zhan X, Fina M, Gobert R, Quan J, Kreutzer M, Arnett S, Hawkins K, Leach A, Tate C, Daniel C, Reyna C, Prince L, Davis S, Purrington J, Bearden R, Weatherly J, White D, Russell J, Sun Q, Tang M, Li X, Scott L, Moresco EM, McInerney GM, Karlsson Hedestam GB, Xie Y, Beutler B (2015). Real-time resolution of point mutations that cause phenovariance in mice. Proc Natl Acad Sci U S A.

[CR332] Wang T, Bu CH, Hildebrand S, Jia G, Siggs OM, Lyon S, Pratt D, Scott L, Russell J, Ludwig S, Murray AR, Moresco EMY, Beutler B (2018). Probability of phenotypically detectable protein damage by ENU-induced mutations in the Mutagenetix database. Nat Commun.

[CR333] Wang W, Corominas R, Lin GN (2019). De novo mutations from whole exome sequencing in neurodevelopmental and psychiatric disorders: from discovery to application. Front Genet.

[CR334] Wang C, Konecki DM, Marciano DC, Govindarajan H, Williams AM, Wastuwidyaningtyas B, Bourquard T, Katsonis P, Lichtarge O (2021). Identification of evolutionarily stable functional and immunogenic sites across the SARS-CoV-2 proteome and greater coronavirus family. Bioinformatics.

[CR335] Wang M, Lee-Kim VS, Atri DS, Elowe NH, Yu J, Garvie CW, Won HH, Hadaya JE, MacDonald BT, Trindade K, Melander O, Rader DJ, Natarajan P, Kathiresan S, Kaushik VK, Khera AV, Gupta RM (2021). Rare, damaging DNA variants in CORIN and risk of coronary artery disease: Insights from functional genomics and large-scale sequencing analyses. Circ Genom Precis Med.

[CR336] Wei P, Liu X, Fu YX (2011). Incorporating predicted functions of nonsynonymous variants into gene-based analysis of exome sequencing data: a comparative study. BMC Proc.

[CR337] Wichmann HE, Kuhn KA, Waldenberger M, Schmelcher D, Schuffenhauer S, Meitinger T, Wurst SH, Lamla G, Fortier I, Burton PR, Peltonen L, Perola M, Metspalu A, Riegman P, Landegren U, Taussig MJ, Litton JE, Fransson MN, Eder J, Cambon-Thomsen A, Bovenberg J, Dagher G, van Ommen GJ, Griffith M, Yuille M, Zatloukal K (2011). Comprehensive catalog of European biobanks. Nat Biotechnol.

[CR338] Willsey AJ, Fernandez TV, Yu D, King RA, Dietrich A, Xing J, Sanders SJ, Mandell JD, Huang AY, Richer P, Smith L, Dong S, Samocha KE, Tourette International Collaborative G, Tourette Syndrome Association International Consortium for G, Neale BM, Coppola G, Mathews CA, Tischfield JA, Scharf JM, State MW, Heiman GA (2017) De novo coding variants are strongly associated with tourette disorder. Neuron 94: 486-499 e9. 10.1016/j.neuron.2017.04.024 10.1016/j.neuron.2017.04.024 PMC5769876 28472652

[CR339] Woolfe A, Mullikin JC, Elnitski L (2010). Genomic features defining exonic variants that modulate splicing. Genome Biol.

[CR340] Worth CL, Preissner R, Blundell TL (2011). SDM–a server for predicting effects of mutations on protein stability and malfunction. Nucleic Acids Res.

[CR341] Wu CH, Apweiler R, Bairoch A, Natale DA, Barker WC, Boeckmann B, Ferro S, Gasteiger E, Huang H, Lopez R, Magrane M, Martin MJ, Mazumder R, O'Donovan C, Redaschi N, Suzek B (2006). The Universal protein resource (UniProt): an expanding universe of protein information. Nucleic Acids Res.

[CR342] Wu S, Tian C, Liu P, Guo D, Zheng W, Huang X, Zhang Y, Liu L (2021). Effects of SARS-CoV-2 mutations on protein structures and intraviral protein-protein interactions. J Med Virol.

[CR343] Xu Q, Tang Q, Katsonis P, Lichtarge O, Jones D, Bovo S, Babbi G, Martelli PL, Casadio R, Lee GR, Seok C, Fenton AW, Dunbrack RL (2017). Benchmarking predictions of allostery in liver pyruvate kinase in CAGI4. Hum Mutat.

[CR344] Yadegari F, Majidzadeh K (2019). In silico analysis for determining the deleterious nonsynonymous single nucleotide polymorphisms of BRCA genes. Mol Biol Res Commun.

[CR345] Yang J, Roy A, Zhang Y (2013). BioLiP: a semi-manually curated database for biologically relevant ligand-protein interactions. Nucleic Acids Res.

[CR346] Yang Y, Peng X, Ying P, Tian J, Li J, Ke J, Zhu Y, Gong Y, Zou D, Yang N, Wang X, Mei S, Zhong R, Gong J, Chang J, Miao X (2019). AWESOME: a database of SNPs that affect protein post-translational modifications. Nucleic Acids Res.

[CR347] Yazar M, Ozbek P (2021). In Silico Tools and Approaches for the prediction of functional and structural effects of single-nucleotide polymorphisms on proteins: an expert review. OMICS.

[CR348] Yin Y, Kundu K, Pal LR, Moult J (2017). Ensemble variant interpretation methods to predict enzyme activity and assign pathogenicity in the CAGI4 NAGLU (Human N-acetyl-glucosaminidase) and UBE2I (Human SUMO-ligase) challenges. Hum Mutat.

[CR349] Yue P, Moult J (2006). Identification and analysis of deleterious human SNPs. J Mol Biol.

[CR350] Yue P, Li Z, Moult J (2005). Loss of protein structure stability as a major causative factor in monogenic disease. J Mol Biol.

[CR351] Yue P, Melamud E, Moult J (2006). SNPs3D: candidate gene and SNP selection for association studies. BMC Bioinformatics.

[CR352] Yue WW, Froese DS, Brennan PE (2014). The role of protein structural analysis in the next generation sequencing era. Top Curr Chem.

[CR353] Zeng Z, Bromberg Y (2019). Predicting functional effects of synonymous variants: a systematic review and perspectives. Front Genet.

[CR354] Zeng S, Yang J, Chung BH, Lau YL, Yang W (2014). EFIN: predicting the functional impact of nonsynonymous single nucleotide polymorphisms in human genome. BMC Genomics.

[CR355] Zeng Z, Aptekmann AA, Bromberg Y (2021). Decoding the effects of synonymous variants. Nucleic Acids Res.

[CR356] Zhang J, Kinch LN, Cong Q, Weile J, Sun S, Cote AG, Roth FP, Grishin NV (2017). Assessing predictions of fitness effects of missense mutations in SUMO-conjugating enzyme UBE2I. Hum Mutat.

[CR357] Zhang X, Li M, Lin H, Rao X, Feng W, Yang Y, Mort M, Cooper DN, Wang Y, Wang Y, Wells C, Zhou Y, Liu Y (2017). regSNPs-splicing: a tool for prioritizing synonymous single-nucleotide substitution. Hum Genet.

[CR358] Zhang T, Hou L, Chen DT, McMahon FJ, Wang JC, Rice JP (2018). Exome sequencing of a large family identifies potential candidate genes contributing risk to bipolar disorder. Gene.

[CR359] Zhang J, Kinch LN, Cong Q, Katsonis P, Lichtarge O, Savojardo C, Babbi G, Martelli PL, Capriotti E, Casadio R, Garg A, Pal D, Weile J, Sun S, Verby M, Roth FP, Grishin NV (2019). Assessing predictions on fitness effects of missense variants in calmodulin. Hum Mutat.

[CR360] Zhou J, Fu BQ (2018). The research on gene-disease association based on text-mining of PubMed. BMC Bioinform.

[CR361] Zhou L, Zhao F (2018). Prioritization and functional assessment of noncoding variants associated with complex diseases. Genome Med.

[CR362] Zhou H, Zhou Y (2002). Distance-scaled, finite ideal-gas reference state improves structure-derived potentials of mean force for structure selection and stability prediction. Protein Sci.

[CR363] Zhou H, Gao M, Skolnick J (2016). ENTPRISE: An Algorithm for Predicting Human Disease-Associated Amino Acid Substitutions from Sequence Entropy and Predicted Protein Structures. PLoS ONE.

[CR364] Zhou H, Gao M, Skolnick J (2018). ENTPRISE-X: Predicting disease-associated frameshift and nonsense mutations. PLoS ONE.

[CR365] Zhou Y, Fujikura K, Mkrtchian S, Lauschke VM (2018). Computational Methods for the Pharmacogenetic Interpretation of Next Generation Sequencing Data. Front Pharmacol.

[CR366] Zlotogora J (2003). Penetrance and expressivity in the molecular age. Genet Med.

[CR367] Zou J, Yin J, Fang L, Yang M, Wang T, Wu W, Bellucci MA, Zhang P (2020). Computational prediction of mutational effects on SARS-CoV-2 binding by relative free energy calculations. J Chem Inf Model.

